# Antibody screening reveals antigenic proteins involved in *Talaromyces marneffei* and human interaction

**DOI:** 10.3389/fcimb.2023.1118979

**Published:** 2023-06-19

**Authors:** Tanaporn Wangsanut, Artid Amsri, Monsicha Pongpom

**Affiliations:** Department of Microbiology, Faculty of Medicine, Chiang Mai University, Chiang Mai, Thailand

**Keywords:** *Talaromyces marneffei*, dimorphic fungus, antigenic proteins, antibody screening, epitopes, membrane proteins, membrane trafficking

## Abstract

Talaromycosis is a fungal infection that generally affects immunocompromised hosts and is one of the most frequent systemic mycoses in HIV patients, especially in endemic areas such as Southeast Asia. *Talaromyces marneffei*, the causative agent of talaromycosis, grows as a mold in the environment but adapts to the human body and host niches by transitioning from conidia to yeast-like cells. Knowledge of the human host and *T. marneffei* interaction has a direct impact on the diagnosis, yet studies are still lacking. The morbidity and mortality rates are high in taloromycosis patients if the diagnosis and treatments are delayed. Immunogenic proteins are excellent candidates for developing detection tools. Previously, we identified antigenic proteins that were recognized by antibodies from talaromycosis sera. Three of these identified proteins have been previously characterized in detail, while the others have not been explored. To expedite the progress of antigen discovery, the complete list of antigenic proteins and their features was fully reported in this study. Functional annotation and Gene Ontology examination revealed that these proteins showed a high association with membrane trafficking. Further bioinformatics analyses were performed to search for antigenic protein characteristics, including functional domains, critical residues, subcellular localization, secretory signals, and epitope peptide sequences. Expression profiling of these antigenic encoding genes was investigated using quantitative real-time PCR. The results demonstrated that most genes were expressed at low levels in the mold form, but were highly upregulated in the pathogenic yeast phase, consistent with the antigenic role of these genes during the human-host interaction. Most transcripts accumulated in the conidia, suggesting a role during phase transition. The collection of all antigen-encoding DNA sequences described here is freely accessible at GenBank, which could be useful for the research community to develop into biomarkers, diagnostic tests, research detection tools, and even vaccines.

## 1 Introduction

*Talaromyces marneffei* is a thermally dimorphic fungus that causes the opportunistic infection, talaromycosis, mostly in immunocompromised patients such as individuals with leukemia, neutropenia, auto-interferon gamma autoantibodies, and AIDS. In fact, in *T. marneffei* endemic areas, infection is very common and has been recognized as a disease indicative of AIDS (Supparatpinyo et al., 1994; Supparatpinyo and Sirisanthana, 1994; Devi et al., 2007). There are over an estimated 8,000 cases of life-threatening *T. marneffei* infections annually with mortality rates of 2-75% (Pal, 2017). Lately, reports of talaromycosis in other immunologically defective patients have been increasing (Chan et al., 2016; Castro-Lainez et al., 2018; Qin et al., 2020; He et al., 2021). *T. marneffei* grows as a saprophytic mold at environmental temperatures (25°C) and undergoes morphological switching to yeast-like cells at human body temperature (37°C) (Boyce and Andrianopoulos, 2013; Boyce and Andrianopoulos, 2015). The route of infection is believed to be through inhalation of the conidia from the air into a patient’s lungs, especially during monsoon season (Chariyalertsak et al., 1996). *T. marneffei* is primarily a pulmonary pathogen, but usually disseminates to other internal organs through lymphatic or hematogenous mechanisms in immunocompromised people (Vanittanakom et al., 2006; Narayanasamy et al., 2021). Overall, critical risk factors for *T. marneffei* infection are related to endemicity, seasons, and immunocompromised conditions. In most cases, the high fatality rate in patients with talaromycosis is mostly associated with delayed diagnosis (Wu et al., 2008; Le et al., 2011).

Host immunity plays an important role in controlling *T. marneffei* infection. Host innate and cellular immunity is involved in the defense against *T. marneffei* because individuals defective in both immunities are predisposed to talaromycosis (Kudeken et al., 1996). Alveolar macrophages are the first innate immune cells first recruited to the site of infection. The macrophages commonly phagocytize and destroy fungal cells by generating reactive oxygen species (ROS) and reactive nitrogen species (RNS) (Gilbert et al., 2014). However, with immunocompromised conditions, *T. marneffei* can survive the macrophage killing and modulate the macrophage to polarize into the M2 type, which triggers an anti-inflammatory response, allowing for tissue repair instead of organism killing (Chen et al., 2015; Dai et al., 2017; Wei et al., 2021; Yang et al., 2021). Then, the infected macrophages serve as a “Trojan host” for dissemination (Ellett et al., 2018). Thus, the ability to survive host immunity contributes significantly to *T. marneffei* virulence, and the mechanisms governing the fungal escape from macrophage killing are well documented (Pongpom et al., 2017; Pruksaphon et al., 2022). The role of humoral immunity (HMI) in *T. marneffei* infection remains unclear. Distinct patterns of immunoreactivity and the presence of IgG antibodies in individual infected serum may correlate with immune impairment severity (Chongtrakool et al., 1997; Vanittanakom et al., 1997). Currently, most of the antigens reacting to antibodies from talaromycosis patients have not been identified and characterized. As a result, it is important to study the host-pathogen interaction, especially the HMI response during *T. marneffei* exposure and infection.

The prevailing gold standard method for detection of talaromycosis is cultivation. Despite its high accuracy, this method is time-consuming, requiring about 1 – 2 weeks to confirm the results. Thus, rapid serodiagnosis, either with antigen or antibody detection is needed. Some serological tests such as enzyme immunoassays for the detection of Mp1p antigen and antibodies have been invented for this purpose. They are commercially available and have proven to be useful for the detection of talaromycosis (Cao et al., 1998; Cao et al., 1999; Wang et al., 2015; Thu et al., 2021; Chen et al., 2022). Additionally, the yeast phase-specific monoclonal antibody 4D1 was successfully used to detect *T. marneffei* infection in human serum and urine samples (Prakit et al., 2016; Pruksaphon et al., 2021). To minimize false negative results associated with available diagnostic tests, a combination of antibody and antigen detection can improve test efficiency. Therefore, the identification of antigenic proteins involved in the *T. marneffei* and human interaction could not only clarify the pathogenesis of mycotic diseases but also could aid in the development of more efficient diagnostic tools.

Immunoproteomics has emerged as a robust tool for antigen identification and has proven effective in screening antigenic proteins from a diverse range of pathogens (Fulton et al., 2019). Notably, it has been applied with success in uncovering antigenic proteins in fungi such as *Aspergillus fumigatus* (Shi et al., 2012; Virginio et al., 2014), *Candida albicans* (Pitarch et al., 2016), *Coccidioides posadasii* (Tarcha et al., 2006a; Tarcha et al., 2006b), *Cryptococcus gattii* (Martins et al., 2013), *Cryptococcus neoformans* (Neuville et al., 2000)*, Paracoccidioides* sp. (Moreira et al., 2019), *Histoplasma capsulatum* (Almeida et al., 2020), and *Sporothrix schenckii* (Rodrigues et al., 2015). However, the progress in the identification of novel antigens in *T. marneffei* has been hampered by a scarcity of studies focusing on this neglected fungal species. Although certain genes have been characterized and demonstrated to possess immunogenic properties (Pongpom et al., 2005; Vanittanakom et al., 2009; Pongpom and Vanittanakom, 2011; Pongpom et al., 2013), the majority of candidate genes have not been extensively investigated. Infact, more than half of the identified proteins remain unexplored. As bioinformatics tools become more advanced and annotated genomes are more accessible, the identification and prediction of both structure and function of unknown sequences are more possible than in the past. Thus, our study focuses on profiling antigenic proteins, specifically emphasizing the previously unreported genes encoding these proteins. Our investigation has yielded a comprehensive collection of antigenic proteins implicated in crucial cellular processes such as stress response, metabolism, and membrane trafficking. We also predicted specific B-cell epitopes for each antigenic protein. Gene expression analysis revealed that these genes are transcriptionally regulated in a phase-specific manner. The information can aid in developing new biomarkers and understanding the interaction between humans and *T. marneffei.*

## 2 Methods

### 2.1 Construction of a bacteriophage lambda expression library

A cDNA library has been constructed in the previous study (Pongpom et al., 2005). Briefly, *Talaromyces marneffei* ATCC 200051 (CBS119456, F4) human isolated strain was cultured in a brain heart infusion broth (BHIB) at 37°C for 3 days. Total RNA was isolated from the yeast cells with TRIzol^®^ reagent (Gibco BRL, Gaithersburg, MD, USA). Then the messenger RNA (mRNA) was isolated with an mRNA purification kit (Oligotex; QIAGEN, Germany). SuperScript^®^cDNA synthesis system (Gibco BRL) was used in the cDNA library construction in the form of lambda bacteriophage.

### 2.2 Purification of immunoglobulin G

The sera were collected from the blood specimens of *T. marneffei*-infected AIDS patients that were sent for clinical analysis at Maharaj Nakorn Chiang Mai hospital. Five sera were pooled, and an immunoglobulin G was purified from the serum by using the HiTrap Protein G HP column (Amersham Pharmacia Biotech, Uppsala, Sweden). The purified immunoglobulin was incubated with *E. coli* Y1090 lysate for 2 hours at 37°C to absorb the antibodies directed to the *E. coli* determinants.

### 2.3 Library screening, purification of the clones, and DNA sequencing

The constructed cDNA library was screened with a purified IgG from a pooled serum. The antibody screening experiment was performed as described previously (Pongpom et al., 2005). The positive phage clones were then isolated and purified by repetition of the antibody screening process until the homogeneous positive signal was generated. Approximately 10^5^ independent plaques from the amplified library were screened with the purified immunoglobulin to obtain immunogenic protein-encoding clones. *E. coli* Y1090 was infected with the phage library and allowed the plaques to grow at 42°C for 4 h. A 50 mM isopropyl-β-D-thiogalactopyranoside (IPTG)-impregnated nitrocellulose membrane (Hybond C-extra; Amersham Pharmacia Biotech) was overlaid on the surface of the culture plate for 3 h at 37°C. The membrane was lifted and then incubated for 4 h at room temperature in a blocking buffer (5% non-fat dry milk, 0.1% Triton X-100 in Tris buffer saline). Then, the membrane was incubated with the purified IgG (25 μg/ml) and HRP-conjugated goat anti-human IgG (20,000-fold dilution in the blocking buffer) for 1 h at room temperature. The antigen-antibody complex was detected by a chemiluminescent substrate (SuperSignal Substrate West Pico; Pierce, Rockford, IL, USA). The positive phage clones were selected and purified by repeated screening until a homogeneous positive signal was generated. Phage-to-plasmid conversion of positive clones was performed by *in vivo* excision using *E. coli* DH10B strain (Gibco BRL) and the insert cDNA were subjected to sequencing by dideoxynucleotide chain termination method.

The DNA sequences of the antigenic protein-encoding clones were deposited in the Genbank database under the following accession numbers: P1; OQ241945, P3; OQ241946, P6; OQ241948, P7; OQ241949, P9; OQ241950, P10; OQ241951, P11; OQ241944, P12; OQ241952, P13; OQ241953, P14; OQ241954, P15; OQ241955, P17; OQ241956, P21; OQ241957, P23; OQ241947, P24; OQ241958, P26; OQ241959, P28; OQ241960.

### 2.4 Functional analysis of the antigenic clones

DNA sequences were submitted to the basic local alignment search tool for nucleotides (BLASTn) to search for sequence similarity against *T. marneffei* strain ATCC18224, and fungal models *Saccharomyces cerevisiae*, *Schizosaccharomyces pombe*, and *Neurospora crassa*. Amino acid sequences of identified antigenic proteins and other fungal homologs were obtained from BLAST search tool for protein (BLASTp). The Clustal program was employed to perform protein sequence alignment. Functional annotation of identified *T. marneffei* proteins was investigated using DAVID server (https://david.ncifcrf.gov/tools.jsp). Functional domains were predicted using Prosite (https://prosite.expasy.org/scanprosite/), SMART (http://smart.embl-heidelberg.de/), and Uniprot (https://www.uniprot.org/) tools. If data prediction was available, STRING (Szklarczyk et al., 2021) was used to analyze the protein-protein interaction network (https://string-db.org/cgi/input.pl), and EggNOG server was used for orthology analysis (Hernández-Plaza et al., 2022) (http://eggnog6.embl.de). The presence of secretory signal sequences and protein subcellular localization were predicted using the SignalP and WoLF PSORT programs (Horton et al., 2007; Teufel et al., 2022). The presence of the proteins within *T. marneffei* extracellular vesicles (EVs) was performed by comparison to available data previously described (Yang et al., 2020). For B-cell linear antigenic epitope prediction, the amino acid sequences were evaluated using the web-based tools Bepipred 2.0, ABCpred, and SVMTriP (Saha and Raghava, 2006; Yao et al., 2012; Jespersen et al., 2017).

### 2.5 Gene expression analysis

*T. marneffei* strain ATCC200051 (CBS119456, F4) was cultured on Sabouraud dextrose agar (SDA) at 25°C for 10-14 days to generate conidia. The conidia were harvested and 1x10^8^ conidia/ml were inoculated in a 50-ml Sabouraud dextrose broth (SDB). Cultures were incubated either at 25°C (mold phase) or 37°C (yeast phase) with continuous shaking at 200 rpm. After 72 hours, cultures were collected by centrifugation at 4°C, 7,000 rpm for 30 min. Total RNA was isolated by using TRIzol^®^ reagent, treated with DNase I, and converted to cDNA as previously described (Amsri et al., 2021). Quantitative real-time PCR was performed using the SYBR Green qPCR mix (Thunderbird SYBR Green Chemistry, TOYOBO). An actin gene was included as a reference gene. All primers used in this study are listed in **Supplemental Table S1**. Calculation of a relative expression was performed using the 2^-(ΔCt)^ where ΔC_t_ = C_t_ actin – C_t_ target). One-way analysis of variance (ANOVA) with Tukey’s multiple comparisons test was performed to test for a significant difference among pairs, using Prism software (GraphPad, version 7.0). Statistical significance was set to *P* < 0.05. Graphs were generated using Prism software. Error bars indicate standard deviation.

Heatmap indicating transcript abundance and expression pattern was generated using the HEATMAP hierarchical clustering web tool (https://www.hiv.lanl.gov/content/sequence/HEATMAP/heatmap.html). The log2 fold-change values of relative expression levels (the 2^-(ΔCt)^ value) were used to construct the heatmap. For visualization purposes, the non-detected expression value of *MPLP6* gene in the conidia and mold phases was adjusted to the lowest gene expression value detected in this analysis.

### 2.6 Construction of Δ*cpeA* and Δ*hsp30 T. marneffei* strains

The construction of Δ*cpeA* and complemented strains were described previously (Pongpom et al., 2013). To generate the Δ*hsp30* mutant, a similar targeted gene deletion approach was performed. Briefly, the deletion construct, containing the *Aspergillus nidulans pyrG* selectable marker flanked by 1.5-kb sequences of 5’ upstream and 3’ downstream regions of the *hsp30* gene (PMAA_014600), was transformed into the *T. marneffei* Δ*ligD*, *pyrG*- strain. The uracil prototroph was selected and the *hsp30* gene deletion was confirmed.

### 2.7 Macrophage killing assay

The human monocytic cell line THP-1 (ATCC TIB-02) was maintained in RPMI-1640 medium containing 10% (v/v) fetal bovine serum (Gibco; Life Technologies, Carlsbad, CA, USA), 100 U penicillin, and 100 mg/ml streptomycin at 37°C with 5% CO_2_. One million cells/well were seeded into a 6-well plate and 100 ng of phorbol myristic acetate (PMA, Sigma, St. Louis, MO, USA) was added to induce macrophage differentiation for 2 days. The macrophage cells were infected with the conidia of Δ*cpeA*, wild type (ATCC18224) and *cpeA* complemented strains at a 10:1 ratio of conidia to macrophages. After 2-h incubation, the cells were washed three times with sterile PBS and lysed with a triton X-100. The fungal cells were harvested by centrifugation, diluted 100 times, and spread onto a potato dextrose agar (PDA). The fungus was cultured at 28°C for 2 days and the colonies were counted and calculated in colony-forming units (CFU). The percentage of fungus killed during macrophage infection was calculated using the equation 100-[(CFUx100)/500,000], in which five hundred thousand represents the baseline control number of conidia not infected by macrophage.

### 2.8 Stress tolerance assays

One thousand conidia harvested from the *T. marneffei* wild type (ATCC18224) and the Δ*hsp*30 mutant were inoculated onto the surface of Aspergillus nidulans minimal medium (ANM) agar containing various stressors. The stress-generated conditions included 1 M sorbitol for osmotic stress, 1 mM hydrogen peroxide (H_2_O_2_) for oxidative stress, and 0.2 M sodium chloride (NaCl) for hyper-salinity stress. The fungal strains were incubated at 37°C for 10 days.

## 3 Results

### 3.1 Screening for antigenic protein-encoding clones

**Figure 1:**
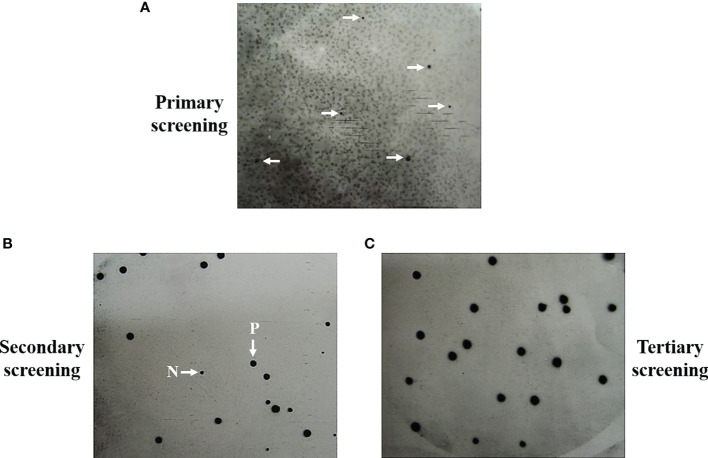
Representation of positive signals from screening and purification of the antigenic protein-encoding clones are shown. Antibody screening was performed on the 10,000 pfu of lambda bacteriophage expression library by using the purified IgG obtained from a pool of *Talaromyces marneffei*-infected serum (n = 5) as the primary antibody. Positive signals were generated from the primary screening **(A)**. The clones obtained from primary plaques were subjected to secondary screening **(B)**. The homogeneous positive signals were obtained in the tertiary screening, showing the purity of the antigenic clones **(C)**. P, Positive plaques. N, Negative plaques.

In previous studies, we identified seventeen *T. marneffei* proteins that exhibited a reaction with antibodies present in the sera of talaromycosis patients (Pongpom et al., 2005). Briefly, an antibody screening experiment was conducted on 10,000 plaque-forming units of the cDNA library to obtain the antigenic protein-encoding genes. *E. coli* Y1090 strain was infected with the bacteriophage lambda library and protein expression was induced. The immunoreaction was performed using purified IgG from sera infected with *T. marneffei* as a probe to detect the antigenic proteins. Chemiluminescent detected distinct positive signals that could be repeated in secondary and tertiary screenings (**Figure 1**) (Pongpom, 2004). Usually, the purified clones were obtained after the second round of immunoreaction. Seventeen positive plaques were purified, and the cDNA inserts were sequenced. Three antigenic protein-encoding genes, *cpeA*, *MPLP6*, and *hsp30* had been previously characterized. They encoded for the catalase-peroxidase (CpeA), MP1-like protein 6 (Mplp6p), and Hsp30 respectively, and they were proven to contain immunogenic properties (Pongpom et al., 2005; Vanittanakom et al., 2009; Pongpom and Vanittanakom, 2011; Pongpom et al., 2013). The remaining fourteen clones in the antigenic protein dataset was subjected to bioinformatic analyses in this current study. The complete sequencing data were deposited at GenBank and are publicly available (**Supplemental Data S1**; See materials and methods).

### 3.2 Bioinformatic analysis of antigenic proteins

The DNA sequences of 14 clones with antigenic properties were subjected to gene identification using the BLAST nucleotide search against the *T. marneffei* strain ATCC 18224. Since the functions of these genes were unknown in *T. marneffei*, we searched for experimentally verified homologous proteins in model yeasts (*Saccharomyces cerevisiae* and *Shizosaccharomyces pombe*) and a model filamentous fungus (*Neurospora crassa*) using BLAST and Uniprot. Results were summarized in **Table 1** and **Supplemental Tables S2–S5**. The following genes were discovered from our original screening result: glutathione peroxidase, NADH-ubiquinone oxidoreductase, 60S ribosomal protein, and stearic desaturase. The others were novel genes whose characteristics have never been previously described (**Table 1**).

**Table 1:** Identification of antigen proteins and putative functions in *T. marneffei*.

Clone ID	Name and accession number of the matched proteins	Possible function
A. Stress responsive proteins		
P1	CpeA Q8NJN2.1	Catalase-peroxidase (oxidative stress response) (Pongpom et al., 2005; Pongpom et al., 2013)
P17	Gpx1 (Hyr1) PMAA_007230	Glutathione peroxidase (oxidative stress response) (Pongpom and Vanittanakom, 2016)
P23	Hsp30 PMAA_014600	Heat shock protein 30 (heat stress response) (Vanittanakom et al., 2009; Yang et al., 2013)
B. Cell wall/cell membrane-associated proteins with secretory signal peptide		
P15	MPLP6 DQ988124.1	Mannoprotein MP1-like protein 6 cell wall-associated protein, adhesin function (Pongpom and Vanittanakom, 2011; Pongpom et al., 2018)
P26	Cell wall protein PMAA_009850	Putative cell-wall protein: containing signal peptide for secretion, unknown function
C. Organelle-membranous proteins (including endosome, vacuole, ER and golgi apparatus)		
P3	Ham13 PMAA_097290	Hyphal fusion protein (hyphal anastomosis related function) (Dettmann et al., 2014)
P6	Mon1 PMAA_029140	Endosome/Vacuolar membrane protein (Meiling-Wesse et al., 2002; Wang et al., 2002; Cabrera et al., 2014)
P7	Fus1 PMAA_067160	Yeast cell fusion protein (Nelson et al., 2004)
P11	Pho88 PMAA_039670	Phosphate transporter, participating in the maturation of secretory proteins, autophagy and mitophagy (Yompakdee et al., 1996; Hurto et al., 2007; Peselj, 2015; Aviram et al., 2016)
P12	Marvel PMAA_006490	Putative cell membrane associated protein, unknown function (MARVEL membrane-associating domain-containing protein) (Sánchez-Pulido et al., 2002)
P21	Nbr1 PMAA_004930	Roles in selective autophagy, mitophagy, pexophagy (Kirkin et al., 2009; Liu et al., 2015; Werner et al., 2019; Zhang et al., 2022)
D. Metabolic process proteins		
P9	Fbp1 PMAA_041280	Fructose-1,6-bisphosphatase (glycolysis/gluconeogenesis)
P10	RPL20A PMAA_054240	60S ribosomal protein L20A (protein translation)
P13	Nuo21.3 PMAA_028280	NADH-ubiquinone oxidoreductase (electron transport chain)
P24	Nmt1 PMAA_034500	Thiamine synthetase (4-amino-5-hydroxymethyl-2-methylpyrimidine phosphate synthase) (thiamine biosynthesis) (Wightman and Meacock, 2003; Nosaka, 2006)
P28	SdeA PMAA_049240	Stearic acid desaturase, Acyl-CoA desaturase 1 (Integral endoplasmic reticulum membrane protein)
P14	Putative C2H2 zinc finger domain protein PMAA_052820	Putative transcription factor containing C_2_H_2_ zinc finger domain

To explore protein functional activities, we utilized ScanProsite and SMART (Simple Modular Architecture Research Tool) tools for functional domain and critical region searches (Schultz et al., 1998; de Castro et al., 2006). Putative protein localization was predicted using Uniprot program. If subcellular compartment prediction for *T. marneffei* proteins was not annotated in Uniprot, we used the protein homologs for the search instead. The web-based tool String was used to search for protein-protein interaction networks and functional enrichment. Lastly, we used the WoLF PSORT and SignalP to identify amino acid sequences for cellular sorting signals.

The antigenic proteins were predicted to localize in five major compartments (**Supplemental Table S2**). First, the protein P14 was predicted to localize in the nucleus, due to its C2H2-zinc finger domain that is commonly found in the transcription factor. Second, several clones were predicted to be cytoplasmic proteins, including fructose-1,6-biphosphatase (Fbp1), ribosomal protein RPL20A, glutathione peroxidase (Gpx1), and thiamine synthase Nmt1. The glutathione peroxidase (Gpx1) was predicted to be located both in the cytosol and the mitochondria. Third, organelles-localizing proteins included the endoplasmic reticulum (ER) membrane proteins Pho88 and stearic acid desaturase SdeA, a vesicular membrane protein Mon1, and a putative mitochondrial inner membrane protein ubiquinone oxidoreductase Nuo21.3. Fourth, the Mplp6p and P26 hypothetical proteins contained signal peptides, suggesting that they might be secretory proteins that localize to the cell wall and excrete extracellularly. Thus, we postulated that Mplp6p and P26 were the cell wall or cell membrane-associated proteins. Lastly, several proteins were predicted to localize in the cell membrane since they possessed the detectable transmembrane portion, or the functional domain associated with the membrane. These membrane-localized proteins were Ham13, Mon1, Fus1, Pho88, Marvel and SdeA.

### 3.3 Epitope prediction of antigenic candidates

**Figure 2:**
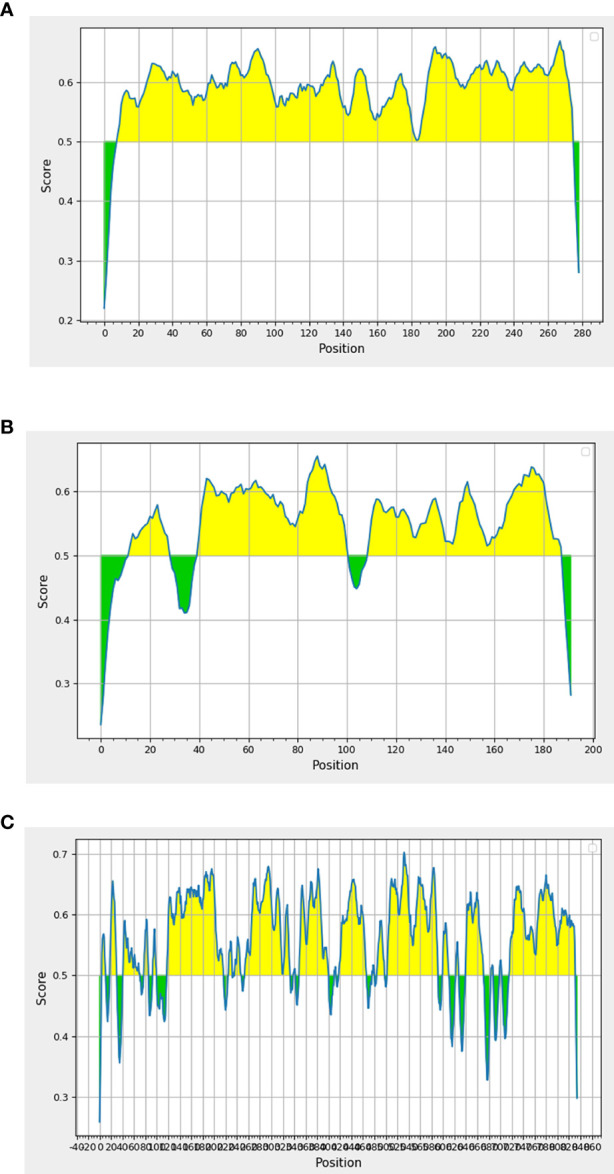
*In silico* analysis of putative epitopes on antigenic protein candidates for the diagnosis of talaromycosis. Results depicted only the top three protein candidates with the highest percentages of epitopes. Antigenic plots were obtained from BepiPred 2.0 server. **(A)** P26, **(B)** Nuo21.3 and **(C)** Nbr1 proteins. Full epitope prediction is described in **Supplemental Data 2**.

Epitope is the specific region of the antigen where the antibody binds. We sought to determine the peptide regions of identified antigens that were potentially recognized by patient antibodies. Therefore, we screened these proteins for putative B-cell epitopes using the bioinformatics analysis approach. As prediction is preferable when overlapped results are obtained from the use of more than one software program (Faria et al., 2011), we mapped putative epitope regions using three different bioinformatic tools (Bepipred, ABCpred, and SVMTriP). All epitope regions obtained from each program and overlapped sequences are presented in **Supplemental Data S2**. All antigenic proteins contained the overlapped epitope regions. The top three antigenic proteins that contained the highest number of predicted epitope peptides are as follows: P26 (Epitope = 95.7%), NADH-ubiquinone oxidoreductase Nuo21.3 (Epitope = 81.8%), and Nbr1 (Epitope = 73.7%) (**Figure 2**). These proteins containing high percentages of B-cell epitopes served as candidate antigens for further investigation.

### 3.4 Gene expression analysis of antigenic proteins

**Figure 3:**
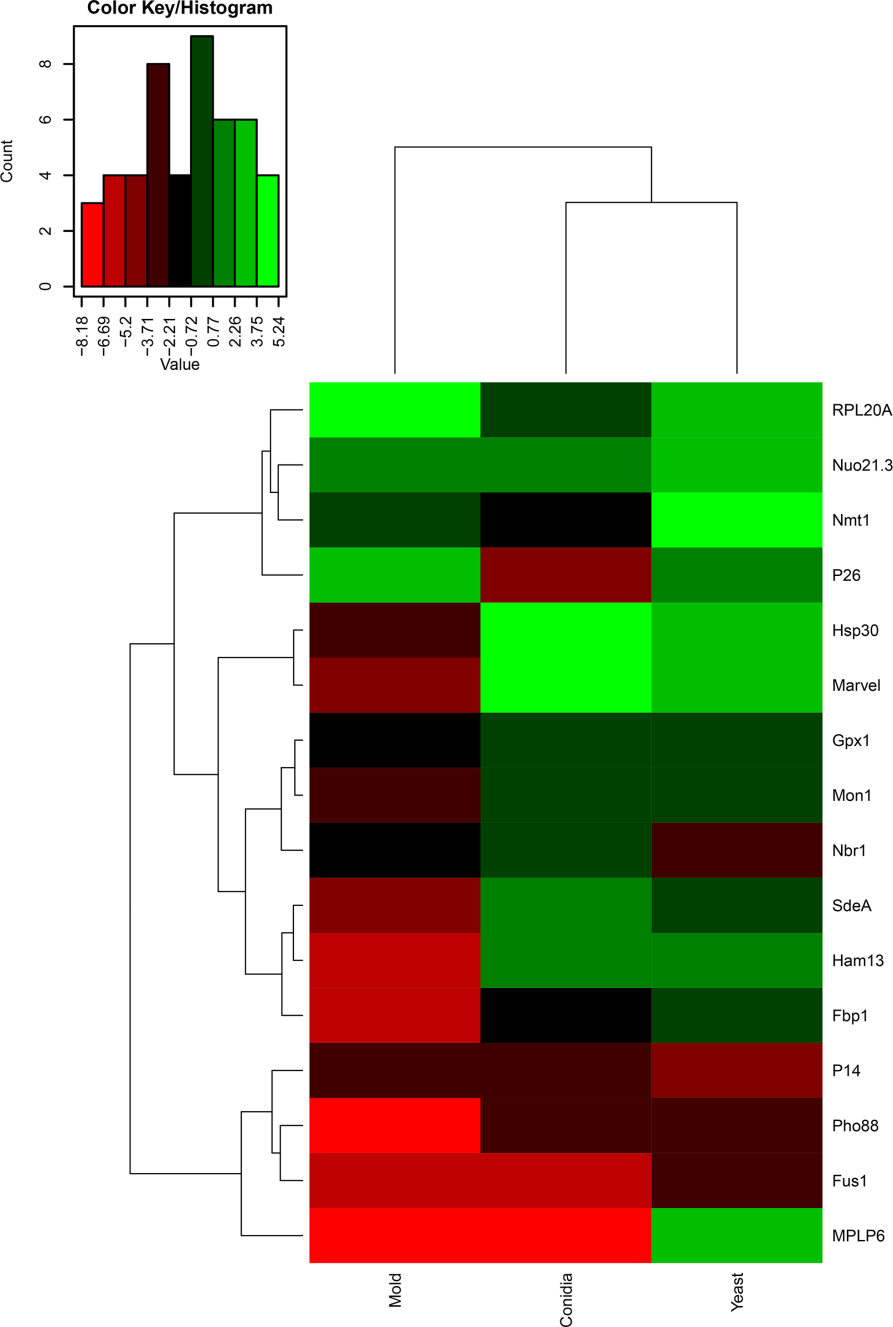
Heatmap depicted gene expression profile of antigenic encoding genes. The names of the antigenic encoding genes are provided on the right side and growth conditions (conidia, mold, and yeast) are provided on the bottom. Dendrograms are on the left and on top of the heatmap. Gene expression was assessed by qRT-PCR when *T. marneffei* was grown in different morphologies. Conidia of *T. marneffei* strain ATCC20051 were inoculated into SDB media and incubated at 25°C (mold) or 37°C (yeast). The 72-h cultures and conidia were harvested, and RNA was prepared (see materials and methods for details). Relative gene expression was calculated by the 2^-ΔCt^ method using actin as a reference gene. Relative fold-change was compared to the phase where the transcript was the lowest. The experiment was performed in three biological replicates. Heatmap was generated using the log2 of relative gene expression values (2^-ΔCt^). The relative gene expression values without the log2 transformation were represented as a bar graph in **Figures 4**, **6**, **7**, **9**–**12** for specified genes.

Changes in gene expression patterns can reflect a change of biological activity or vice versa. To better understand the function of identified genes, we assessed the gene expression profiles of all antigenic encoding genes. Since dimorphism is an essentially biological process of *T. marneffei*, required for the virulence of this fungal pathogen. We reasoned that the identification of phase-specific proteins would be beneficial for developing detection tools in both clinical and research settings. Our antigenic protein screening experiment was performed with *T. marneffei* growing in only the yeast phase, and therefore we wondered if the expression levels of these genes would change with *T. marneffei* in different morphologies. To investigate the gene expression profiles of identified genes, conidia from the *T. marneffei* were either directly harvested or inoculated into Sabouraud’s dextrose broth and grown for 3 days at 25°C or 37°C to induce either the mold or yeast phase, respectively. RNA was collected from these samples, and quantitative real-time PCR was performed to assess transcript levels. A heat map was generated using log2 values of gene expression data (2^-ΔCt^), so that we could compare both (i) gene expression fold-change and (ii) transcript abundance across genes and phases (**Figure 3**; **Supplemental Table S6**). The color scheme assigned green to genes with the highest abundance and red to genes with the lowest expression. In addition, hierarchical analysis was performed to group genes with similar expression profiles.

**Figure 4:**
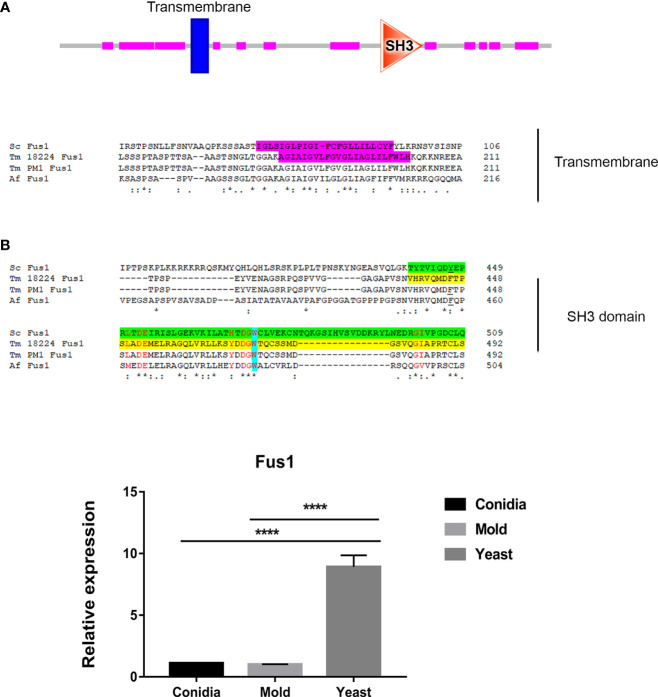
The antigenic protein shows high homology with Fus1 protein from *S. cerevisiae* and other fungal species. **(A)** Sequence alignment of Fus1 and homologous proteins are shown. Protein domain prediction tools reveal that Fus1 contains the conserved Src Homology 3 (SH3) domain and a transmembrane region. The transmembrane region is highlighted in magenta. The SH3 domain is shown in green (ScFus1) and yellow (TmFus1). The conserved tryptophan residue is shown in blue. Residues constituting the conserved surface regions are shown in red font. EggNOG Gene orthology analysis reveals that Tm Fus1 and its orthologs function in signal transduction mechanism (ENOG4103SS1). **(B)** Fus1 gene expression was assessed when *T. marneffei* was grown in yeast, mold, or conidia. Strains were grown, RNA was prepared, and gene expression was analyzed as described in the legend of **Figure 3**. The experiment was performed in three biological replicates. Error bars indicate standard deviation. Statistically significant values (**** *P*≤ 0.0001) are indicated. Protein sequence alignment was performed using Clustal omega from web-based tool analysis https://www.ebi.ac.uk/Tools/msa/clustalo/). Protein domains were identified using ScanProsite and SMART tools (https://prosite.expasy.org/scanprosite/; http://smart.embl-heidelberg.de).

**Figure 5:**
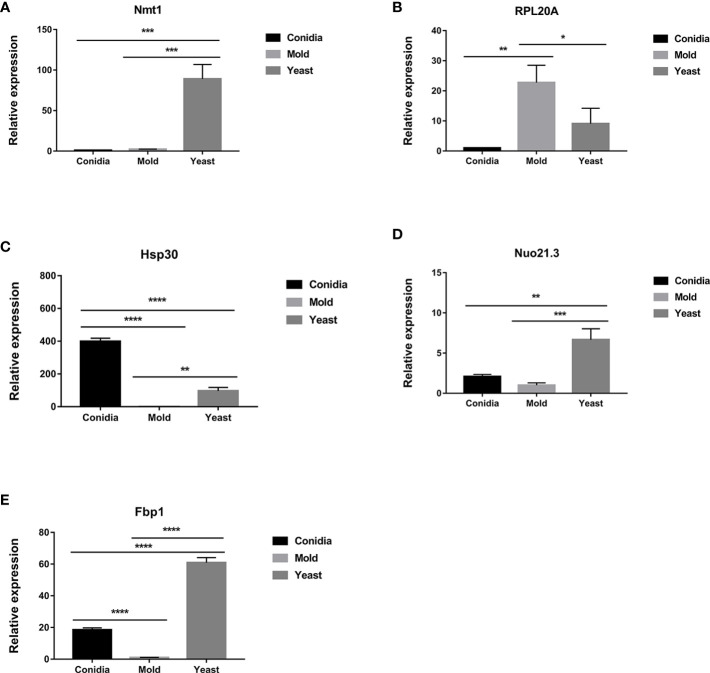
The transcript levels of antigen-encoding genes are differentially expressed when cultured in different morphologies. Expression levels of **(A)** *nmt1*, **(B)** *RPL20*A, **(C)** *hsp30*, **(D)** *Nuo21.3*, and **(E)** *fbp1* are shown. Strains were grown, RNA was prepared, and gene expression was analyzed as described in the legend of **Figure 3**. The experiment was performed in three biological replicates. Error bars indicate standard deviation. Statistically significant values (* *P*≤ 0.05, ** *P*≤ 0.01, *** *P*≤ 0.001, **** *P*≤ 0.0001) are indicated.

**Figure 6:**
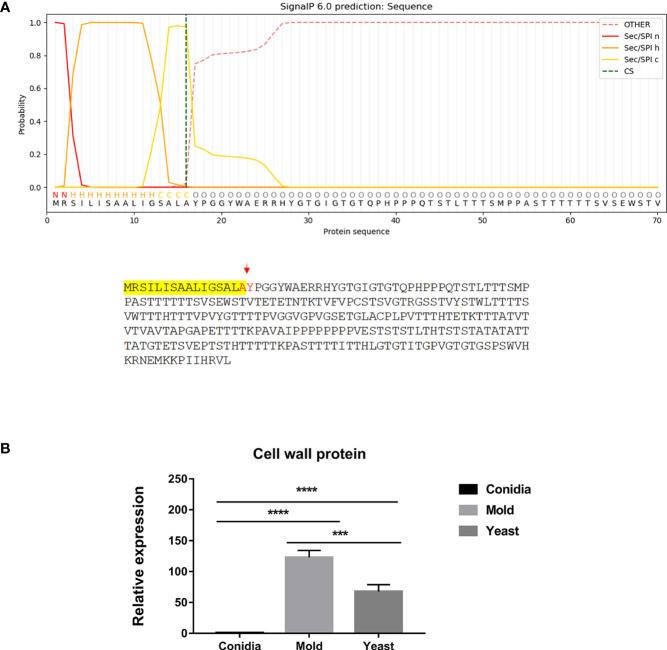
The antigenic clone P26 is predicted to contain a signal peptide and likely enters the secretory pathway. **(A)** The signal peptide is located at the N-terminus and is highlighted in yellow. Predicted cleavage residues are indicated in red. Protein subcellular compartment, signal peptide, and cleavage site were predicted using the web-based tools as follows: https://services.healthtech.dtu.dk/service.php?SignalP; https://www.genscript.com/wolf-psort.html. **(B)** The P26 gene expression was analyzed in *T. marneffei* grown in yeast, mold, or conidia. Strains were grown, RNA was prepared, and gene expression was analyzed as described in the legend of **Figure 3**. The experiment was performed in three biological replicates. Error bars indicate standard deviation. Statistically significant values (*** *P*≤ 0.001, **** *P*≤ 0.0001) are indicated.

**Figure 7:**
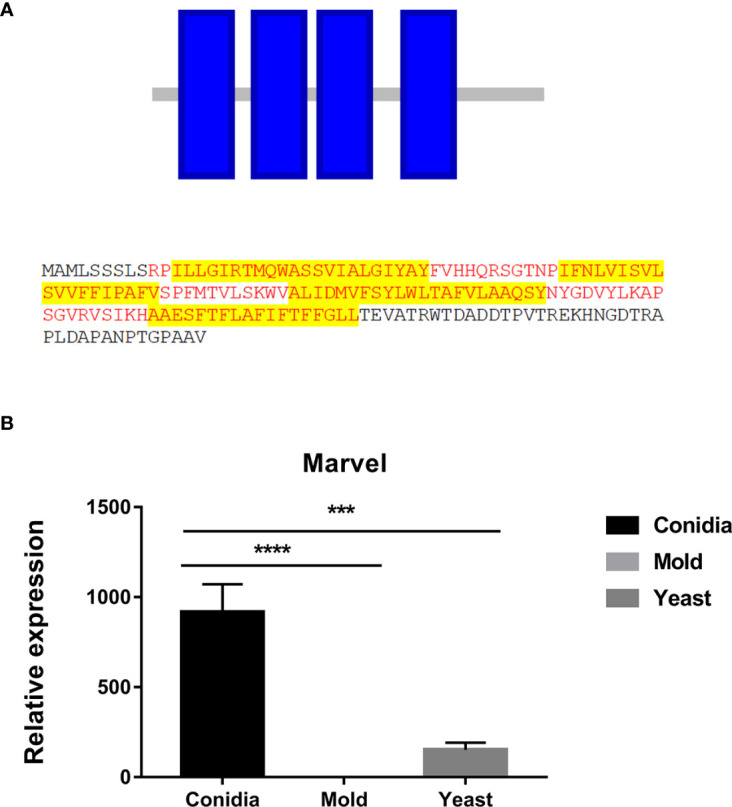
The antigenic protein is predicted to contain the Marvel membrane-associating domain. **(A)** The identified protein contained 4 putative transmembranes (blue box) and was predicted to be an integral membrane protein. Putative sequences for the marvel domain are highlighted in red fonts. Highlights in yellow depict transmembrane sequences. Protein domains were identified using ScanProsite and SMART tools (https://prosite.expasy.org/scanprosite/; http://smart.embl-heidelberg.de). **(B)** TmMarvel gene expression was analyzed when *T. marneffei* was grown in yeast, mold, or conidia. Strains were grown, RNA was prepared, and gene expression was analyzed as described in the legend of **Figure 3**. The experiment was performed in three biological replicates. Error bars indicate standard deviation. Statistically significant values (*** *P*≤ 0.001, **** *P*≤ 0.0001) are indicated.

As shown in **Figure 3**, the genes that exhibited yeast-phase-specific expression were *MPLP6* and *Fus1*. As a control, the *MPLP6* transcript was not detected in either mold or conidia, consistent with previous studies (Pongpom and Vanittanakom, 2011). The *Fus1* transcript was significantly upregulated by 9-fold in the yeast phase and expressed at very low levels in the mold and conidial phases (**Figures 3**, **4B**). Furthermore, the *Nmt1* gene was differentially upregulated in the yeast phase, exhibiting almost a 90-fold increase in the yeast sample (**Figures 3**, **5A**). Notably, the *Nmt1* transcript in the yeast phase was one of the highest abundant transcripts. We did not detect any genes that were exclusively expressed in the mold phase. However, *RPL20A* (**Figures 3**, **5B**) and *P26* (**Figures 3**, **6B**) genes showed the highest fold change increase in the mold form, upregulating by 23- and 123-fold, respectively. We noted that the *RPL20A* and *P26* genes were partially upregulated in the yeast phase (**Figures 3**, **5B**, **6B**). Although none of the identified genes were specifically expressed in conidia, we found that Marvel (**Figures 3**, **7B**) and *hsp30* (**Figures 3**, **5C**) genes were highly expressed in conidia. These two genes were also genes that exhibited the highest fold change in each phase. The Marvel gene was upregulated by 900-fold and 152-fold, and the *hsp30* gene was upregulated by 400-fold and 96-fold in the conidia and yeast forms, respectively. Lastly, our gene expression analysis revealed that *RPL20A* and *Nuo21.3* showed the highest transcript abundance among identified genes (**Figure 3**).

For those reported genes, our current gene expression profile was consistent with previously published data. For example, expression levels of *hsp30* (**Figure 5C**), *Nuo21.3* (**Figure 5D**), and *fbp1* (**Figure 5E**) genes showed high induction in the conidia and yeast phases (Pongpom and Vanittanakom, 2016). Only the *gpx1* gene showed a different expression profile compared to previous reports, which could be attributed to variations in media.

### 3.5 Antigenic proteins are related to the membrane trafficking pathway

**Figure 8:**
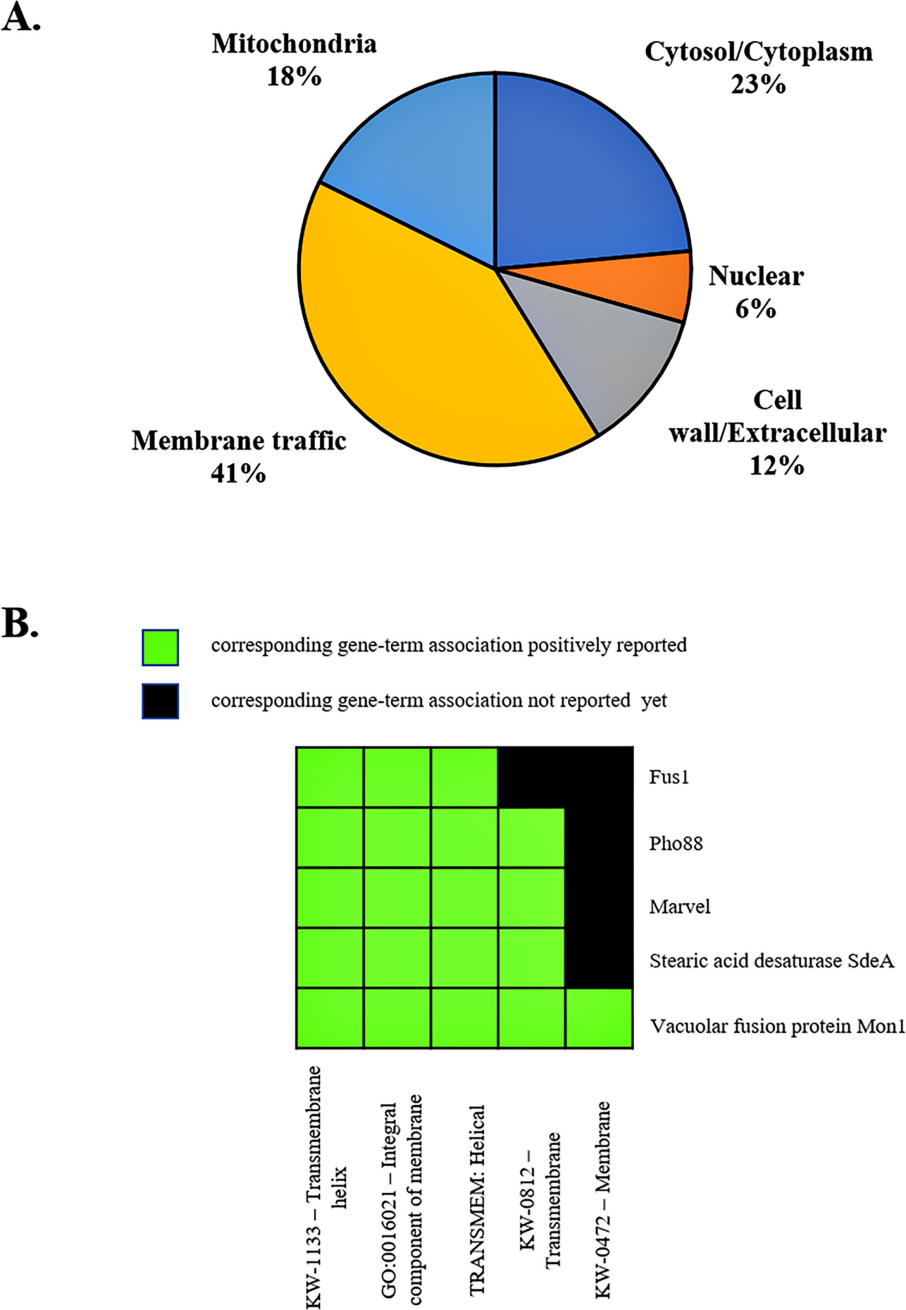
Subcellular localization and functional annotation clustering of antigenic proteins indicates a membrane-associated role. **(A)** The pie chart depicts the subcellular localization of identified antigenic proteins. Membrane traffic describes proteins localized to the membrane, endosome, vacuole, vesicle membranes, ER, and the Golgi apparatus. Subcellular compartments of proteins are based on Uniprot database and WoLF PSORT servers, using protein ID from *T. marneffei* and fungal homologs. **(B)** A 2D view of functional annotation clustering is shown. Biological function and enrichment pathways were analyzed using the DAVID bioinformatic tool.

The membrane trafficking system is crucial for maintaining proper cargo distribution and compartmentalization within cells, as well as for facilitating autophagy, a cellular degradation process (Yang and Rosenwald, 2014). In autophagy, a double-membrane structure is formed and then fused with the lysosome or vacuole for hydrolysis and recycling of building blocks. We noticed that the majority of identified antigenic proteins (53%) were predicted to localize in the cell membrane, membrane-bound organelles, and extracellular compartment (**Figure 8A**; **Supplemental Table S2**). Also, some proteins had an overlapping role in autophagy (**Table 1**). To better understand the function of the antigenic proteins, Gene Ontology (GO) enrichment and putative biological functions of all identified genes were investigated using the DAVID server. Strikingly, there were many antigenic proteins that showed enrichment in membrane-related functions (**Figure 8B**; **Table 1**; **Supplemental Tables S3**, **S5**). Thus, we hypothesized that these proteins function in the membrane trafficking pathway and play a role in antigen presentation during *T. marneffei* infection inside the macrophages. The relevance of antigenic proteins or their homologous proteins to the membrane trafficking system was highlighted, as described below.

#### 3.5.1 TmMon1

**Figure 9:**
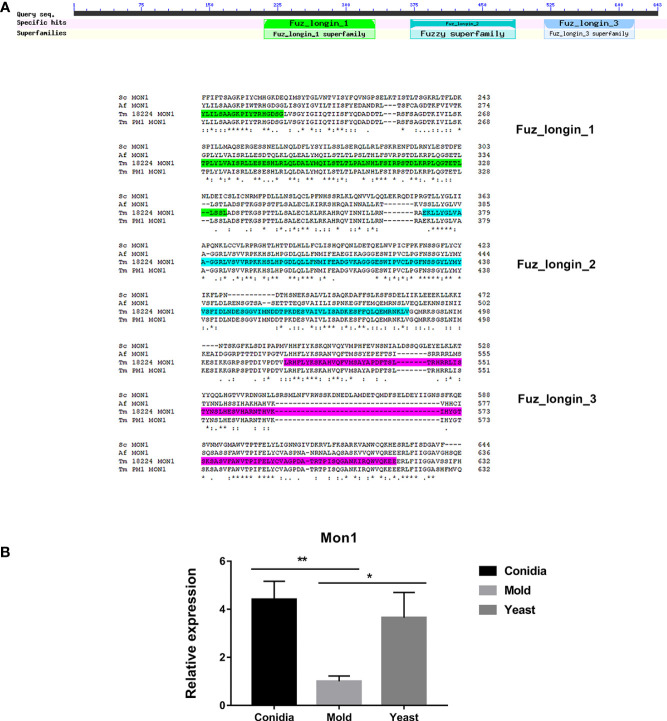
Mon1 protein sequence alignment shows high homology with other fungal Mon1 proteins. **(A)** The Tm Mon1 contains three of the conserved longin domains. Protein sequence alignment was performed using the web-based tool analysis Clustal Omega available at https://www.ebi.ac.uk/Tools/msa/clustalo/. BLAST analysis tool was used to identify fungal homologs. Full protein IDs are as follows: Sc Mon1 = NP_011391.2 from *Saccharomyces cerevisiae*; Af Mon1 = KAH1333016.1 from *Aspergillus fumigatus*; Tm Mon1 = XP_002144612.1 from *T. marneffei* strain ATCC 18224; Tm Mon1 = KFX53411.1 from *T. marneffei* strain Pm1. **(B)** Mon1 gene expression was assessed by qRT-PCR when *T. marneffei* was grown in different morphologies. Strains were grown, RNA was prepared, and gene expression was analyzed as described in the legend of **Figure 3**. The experiment was performed in three biological replicates. Error bars indicate standard deviation. Statistically significant values (* *P*≤ 0.05, ** *P*≤ 0.01) are indicated.

In *S. cerevisiae*, Mon1 is a subunit of the heterodimeric guanine nucleotide factor (GEF) Mon1-Ccz complex. This Mon1-Ccz complex stimulates nucleotide exchange and activation of Ypt7, a Rab GTPase, thereby marking endosomes and autophagosomes for fusion with lysosomes/vacuoles and degradation (Klink et al., 2022). Thus, Mon1 is required for Ypt7 localization to the vacuolar membrane, vacuolar fusion, and autophagy. Gene orthology analysis using EggNOG database suggests that TmMon1 and its orthologs function in intracellular trafficking, secretion, and vesicular transport (ENOG4103JWM). Proteins in the Rab-GEF family universally contain the conserved tri-Longin domain. Longin is a subtype of SNARE, the protein superfamily required for intracellular membrane fusion function (Filippini et al., 2001; Rossi et al., 2004). As seen in **Figure 9A**, TmMon1 contains three of the conserved longin domains. To predict the TmMon1 interaction network, the amino acid sequences of TmMon1 were subjected to STRING analysis. The predicted interacting partners of TmMon1 showed enrichment in the vacuolar trafficking process (**Supplemental Figure S1**). Altogether, bioinformatics analyses strongly suggest the role of TmMon1 in regulating membrane fusion in *T. marneffei*.

To assess the TmMon1 gene expression profile, we performed quantitative real-time PCR. We found that TmMon1 showed increased transcript in the conidia and yeast forms, exhibiting 4-fold and 3-fold, respectively, higher than the mold form (**Figure 9B**). This result suggests an important function of TmMon1 in the germination stage and during yeast survival.

#### 3.5.2 TmFus1

In *S. cerevisiae*, Fus1 is the plasma membrane protein required for septum degradation during the cell fusion process (Trueheart et al., 1987; Bagnat and Simons, 2002; Nelson et al., 2004). Fus1 acts as a scaffold for the assembly of signaling and polarity proteins, facilitating polarized secretion of vesicles containing enzymes responsible for septum-degrading (Gammie et al., 1998).

Fus1 from *S. cerevisiae* contains the C-terminal Src Homology 3 (SH3) domain. The SH3 domain mediates protein-protein interaction, and the conserved residue Trp (W36) plays a key role in the binding reactions of almost all SH3 domains (Kim et al., 2008). The SH3 domain-containing proteins play versatile and diverse roles in the cells such as altering the subcellular localization of signaling pathway components or facilitating multiprotein complex formation. As shown in **Figure 4A**, the TmFus1 contains transmembrane and SH3 domains, implying the role of TmFus1 at the plasma membrane. The conserved residue tryptophan of the SH3 domain is highly conserved in TmFus1 (**Figure 4A**). Gene orthology analysis revealed that TmFus1 and the function of its orthologs in signal transduction mechanisms (ENOG4103SS1) are consistent with the integral role of membrane trafficking and signaling pathways (Gonnord et al., 2012).

As mentioned previously, the TmFus1 gene expression exhibited a yeast-phase-specific profile (**Figure 4B**). The upregulation of the TmFus1 transcript during yeast growth suggested that *T. marneffei* might undergo events associated with cell fusion or signaling during host infection.

#### 3.5.3 TmHam13

In filamentous fungus *N. crassa*, cell-to-cell communication and cell fusion are fundamental biological processes, occurring in both the sexual cycle and vegetative growth. The fusion of conidial germlings contributes to the earliest steps of colony establishment while the fusion of hyphae (anastomosis) increases the interconnectedness of the mycelial network (Dettmann et al., 2014; Fischer and Glass, 2019). Many “hyphal anastomosis mutant” (ham) genes have been previously identified (Xiang et al., 2002; Fu et al., 2011; Fischer and Glass, 2019). These genes are mostly involved with vesicular trafficking, membrane fusion, signal transduction, and transcription factors. Ham13 has been characterized as a gene necessary for germling communication, signaling transduction and cell fusion (Dettmann et al., 2014).

**Figure 10:**
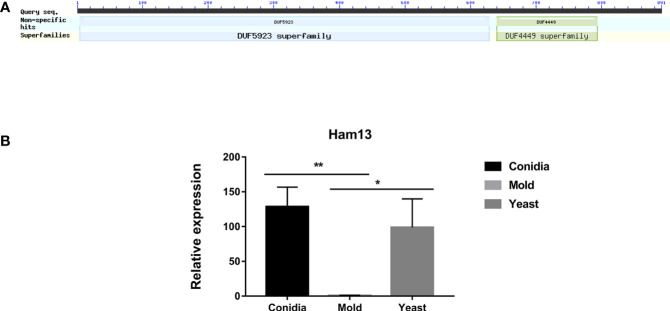
The antigenic protein shows high homology with the hyphal anastomosis Ham-13 protein. **(A)** Ham-13 protein contains two fungal conserved domains with unknown functions, DUF4449 and DUF5923. In filamentous fungi *N. crassa*, Ham-13 plays an important role in cell-cell communication and hyphal anastomosis (hyphal fusion) in MAK-2 kinase dependent manner. A detail of protein sequence alignment is depicted in **Figure S2**. **(B)** Ham13 gene expression was analyzed when *T. marneffei* was grown in yeast, mold or conidia. Strains were grown, RNA was prepared, and gene expression was analyzed as described in the legend of **Figure 3**. The experiment was performed in three biological replicates. Error bars indicate standard deviation. Statistically significant values (* *P*≤ 0.05, ** *P*≤ 0.01) are indicated.

TmHam13 showed high homology with Ham13 from *N. crassa* and SPAC32A11.02c gene from *S. pombe*. It contains two functional domains DUF4449 and DUF5923 (**Figure 10A**). These domains of unknown functions are highly conserved among compared proteins (**Figure S2**). We investigated the gene expression profile of TmHam13 in relation to growth phases. TmHam13 gene was highly upregulated in the conidia and yeast phases, exhibiting 128-fold and 99-fold, respectively higher than the mold form (**Figure 10B**). This result suggests the important role of this TmHam13 gene in conidial and yeast growth, likely related to cell fusion and signaling transduction events during pathogenesis.

#### 3.5.4 TmNbr1

**Figure 11:**
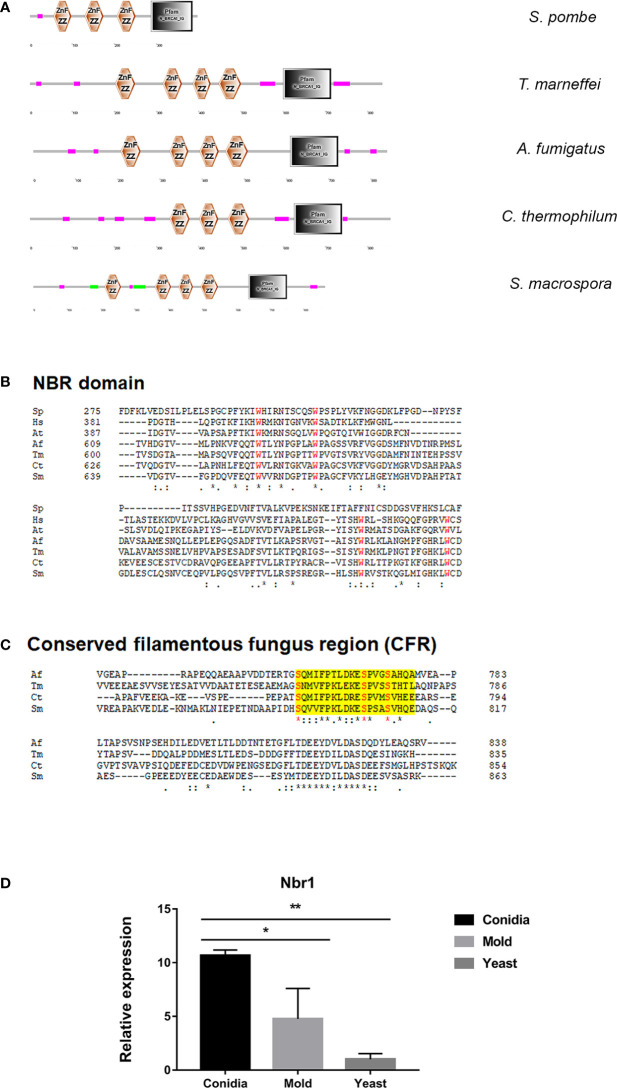
The antigenic protein shows high homology with the conserved autophagy receptor Nbr1. **(A)** The protein architecture of Nbr1 homologs in fungal species is shown: Magenta box = low complexity region; Green box = coiled-coil region; Black box = N_BRCA1_IG (Ig-like domain from next to BRCA1 gene, PF16158); Znf ZZ = ZZ-type zinc finger domain. **(B)** Alignment of the Nbr domain of the following species: Sp = *S. pombe* (NP_001342955.1); Hs = *Homo sapiens* (Q14596.3); At = *Arabidopsis thaliana* (OAO97459.1); Sm = *Sordaria macrospora* (XP_003346367.1); Ct = *Chaetomium thermophilum* (XP_006696593.1); Tm = *T. marneffei* (XP_002152652.1); Af = *A*. *fumigatus* (XP_755022.1). Red font highlights the four conserved tryptophan (FW) residues typical for this Nbr1 domain. **(C)**. The conserved fungal region (CFR) at the C-terminal was found in Tm Nbr1 and other filamentous ascomycetes. Yellow shading depicts the CFR, and red font highlights the 3 conserved serine (S) residues. **(D)** TmNbr1 gene expression was analyzed in *T. marneffei* grown in yeast, mold, or conidia. Strains were grown, RNA was prepared, and gene expression was analyzed as described in the legend of **Figure 3**. The experiment was performed in three biological replicates. Error bars indicate standard deviation. Statistically significant values (* P≤ 0.05, ** P≤ 0.01) are indicated.

In autophagy, cytoplasmic materials can be transported to lysosomes/vacuoles in a non-selective or a selective manner (Feng et al., 2014; Ohsumi, 2014; Stolz et al., 2014; Liu et al., 2015; Gatica et al., 2018; Zhao et al., 2020). In selective autophagy, the Neighbor of BRAC1 (Nbr1) is an autophagy cargo receptor conserved across eukaryotes, but notably absent in *S. cerevisiae*. In the fission yeast *S. pombe*, Nbr1 transports degrading enzymes in the specific Nbr1-mediated vacuolar targeting pathway (NVT) (Liu et al., 2015; Wang et al., 2021). As shown in **Figure 11A**, the putative cargo receptor TmNbr1 of *T. marneffei* displays a similar domain architecture as Nbr1 homologs from other eukaryotes, especially filamentous fungi. Nbr1 proteins contain a variable number of domains in different eukaryotic species, but commonly include the ZZ-type zinc-finger domain and the Nbr1 domain (Werner et al., 2019). TmNbr1 contains 4 copies of the ZZ-type zinc-finger domain, similar to the Nbr1 homologs from filamentous fungi *A. fumigatus, Chaetomium thermophilum* and *Sordaria macrospora*. The four conserved tryptophan residues (FW) are a signature domain of Nbr1, playing an important role in protein binding and cargo recognition (Zhang et al., 2022). The FW domains become degenerated in the fission yeast Nbr1 (Zhang et al., 2022). Accordingly, the four tryptophan residues are highly conserved in the TmNbr1 (**Figure 11B**). Additionally, the conserved fungal region (CFR) is a characteristic of filamentous ascomycetes, yet not present in yeasts, plants, or mammals. The CFR region contains three conserved serine residues located near the Nbr1 domain. As seen in **Figure 11C**, the CFR and key residues are conserved in TmNbr1, suggesting that CFR is found not only in filamentous ascomycetes but also in dimorphic fungi. To predict the TmNbr1 interaction network, the amino acid sequences of TmNbr1 were subjected to STRING analysis. The predicted interacting partners of TmNbr1 showed enrichment in autophagy-related and ubiquitination-related processes (**Figure S3**). Altogether, bioinformatics analyses strongly suggest the role of TmNbr1 in autophagy via the NVT pathway.

To examine the gene expression pattern of TmNbr1, we performed the quantitative real-time PCR to measure its transcript levels in the conidia, yeast, and mold phases. We found that TmNbr1 is expressed at low levels in the yeast phase. The TmNbr1 is upregulated 11- and 5-fold in the conidia and mold forms, respectively (**Figure 11D**). This result suggests that TmNbr1 likely plays a critical role in conidia germination and mold growth.

#### 3.5.5 TmPho88 (Snd3)

In *S. cerevisiae*, Pho88 was originally identified as a membrane-associated protein, localized to ER, and involved in the inorganic phosphate transport and the maturation of secretory proteins (Yompakdee et al., 1996; Hurto et al., 2007; Copic et al., 2009). In addition, Pho88 is necessary for efficient autophagy and mitophagy (the selective degradation of mitochondria by autophagic machinery) (Peselj, 2015). A later mutant screening study identified Pho88 as one of the SND proteins, functioning in the Signal Recognition Particle-iNDependent pathway (SND pathway) for targeting a broad range of substrate proteins to the ER (Aviram et al., 2016). The SND pathway works in parallel with the ER targeting SRP and GET (Guided Entry of Tail-anchored proteins) pathways (Walter and Johnson, 1994; Rapoport, 2007; Stefanovic and Hegde, 2007; Favaloro et al., 2008). Altogether, Pho88 plays a strong important role in multiple membrane trafficking-related pathways.

**Figure 12:**
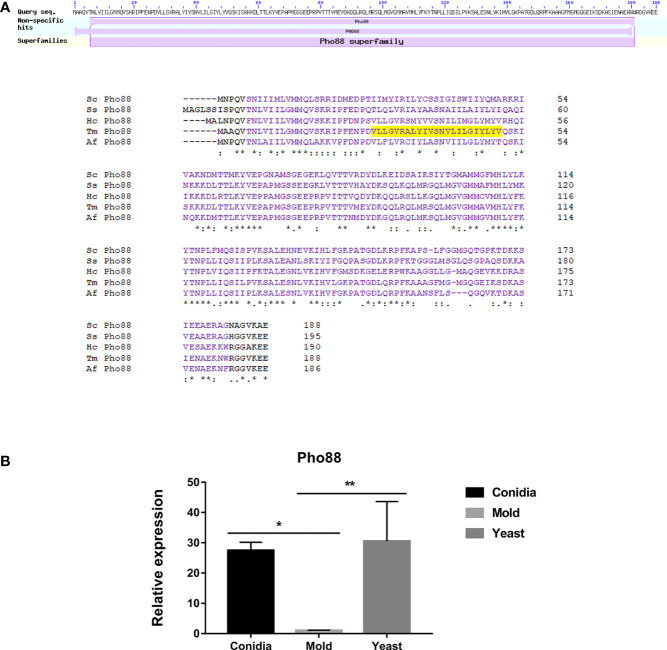
The antigenic protein shows high homology with other fungal Pho88 proteins. **(A)** Pho88 contains a transmembrane and the Pho88 conserved domain. In *S. cerevisiae*, Pho88 plays a role in the maturation of secretory proteins and is required for efficient autophagy and mitophagy. It mainly localizes in the endoplasmic reticulum. Also, its role is involved with the inorganic phosphate transport pathway. The transmembrane region is shown in shaded yellow. Protein sequence alignment was performed using the web-based tool analysis Clustal Omega available at https://www.ebi.ac.uk/Tools/msa/clustalo/. BLAST analysis tool was used to identify fungal homologs. Full protein IDs are as follows: Sc Pho88 = NP_009664.1 from *Saccharomyces cerevisiae*; Af Pho88 = XP_748146.1 from *Aspergillus fumigatus*; Tm Pho88 = XP_002151103.1 from *T. marneffei* strain ATCC 18224; Ss Pho88 = ERS95482.1 from *Sporothrix schenckii* ATCC 58251*;* Hc Pho88 *=* EGC50042.1 from *Histoplasma capsulatum* H88. **(B)** Pho88 gene expression was evaluated in *T. marneffei* grown in yeast, mold, or conidia. Strains were grown, RNA was prepared, and gene expression was analyzed as described in the legend of **Figure 3**. The experiment was performed in three biological replicates. Error bars indicate standard deviation. Statistically significant values (* *P*≤ 0.05, ** *P*≤ 0.01) are indicated.

As shown in **Figure 12A**, the protein sequence alignment of TmPho88 showed high homology with the Pho88 proteins from other fungal species. TmPho88 contains the conserved Pho88 domain and one copy of the transmembrane, consistent with its predicted role as a membrane protein. Gene expression analysis by quantitative real-time PCR demonstrates that TmPho88 is highly expressed in conidia and yeast growth. The TmPho88 gene is expressed at a 27-fold increase in conidia and at a 31-fold increase in the yeast phase when compared to growth in mold morphology (**Figure 12B**). Thus, TmPho8 is predicted to function in phosphate transport, ER targeting, and autophagy, especially during conidia germination and yeast survival in the human host.

### 3.6 Proteins with unknown experimentally verified homologs, but containing sequences related to membrane trafficking

There are several identified proteins that contain functional domains or regions related to membrane trafficking functions. However, the homologs of these proteins have not been experimentally characterized in any fungal model species, thus the actual functions have not been validated. First, the MARVEL-domain containing protein (TmMarvel) was found in our screening result. MARVEL (MAL and related proteins for vesicle formation and membrane link) is a conserved domain that is comprised of four transmembrane helices (Sánchez-Pulido et al., 2002). The function of this domain is to mediate protein-lipid interaction, which is required for the localization or formation of diverse specialized membrane subdomains (Yaffe et al., 2012; Rubio-Ramos et al., 2021). The TmMarvel-encoding gene (PMAA_006490) gives two different transcripts, the first one encoding the 173-amino acid protein (TmMarvelA) with four transmembrane helices (**Figure 7A**), and the other encoding 125-amino acid protein (TmMarvelB) with only three transmembrane helices (data not shown). As mentioned in the previous section, gene expression profiling revealed that the expression of the TmMarvel encoding gene was drastically upregulated in the conidia and yeast phases, increasing by 900-fold and 152-fold, respectively (**Figure 7B**). Collectively, TmMarvel was likely upregulated in conidia and yeast to mediate specific membrane apposition events.

Second, the antigenic clone P26 contained the signal peptide, which is the short N-terminal amino acid sequence that targets proteins to the secretory pathway in eukaryotes. As shown in **Figure 6A**, the P26 protein contains the signal peptide with good predictive value (signal probability, 0.9998; SignalP 6.0) (Teufel et al., 2022). The protein localization was predicted using WoLF PSORT (Horton et al., 2007), and the result indicated that P26 protein contained the ER membrane retention signals (XXRR-like motif), RSIL, in the N-terminus (**Figure 6A**), and it might localize extracellularly (predictive values; 55.6%: extracellular, including cell wall, 22.2%: mitochondrial, 11.1%: nuclear, and 11.1%: cytoplasmic). Gene expression analysis revealed that P26 encoding gene was highly upregulated in both the mold and yeast phases. Its expression was elevated by 123- and 68-fold in the mold and yeast phases, respectively (**Figure 6B**). This result suggests that the P26 protein likely enters the secretory pathway, necessary for both mold and yeast morphology.

### 3.7 Phenotypic characterization of identified immunogens during the stress response

**Figure 13:**
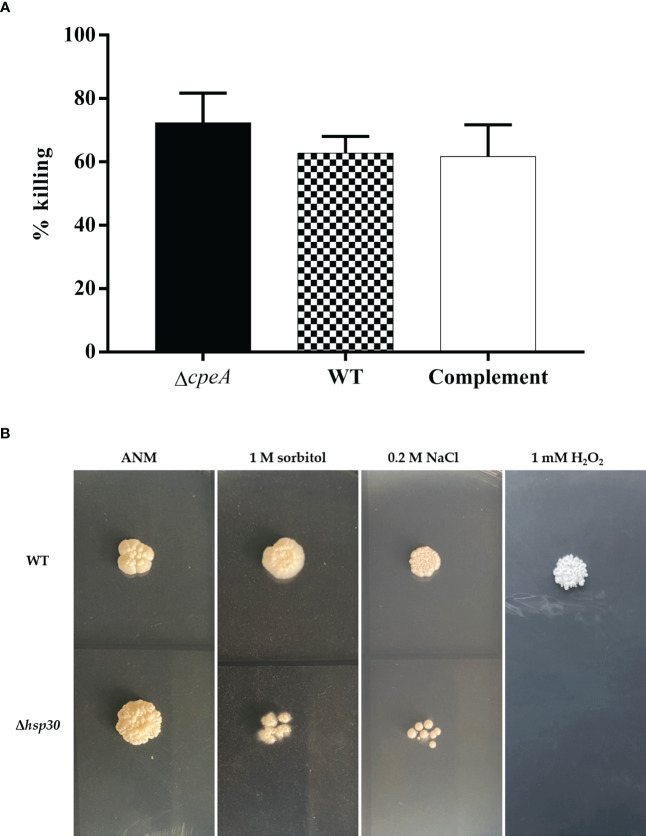
Phenotypic characterization was performed in *T. marneffei* lacking immunogenic genes. **(A)** Macrophage killing assay was used to determine the involvement of *cpeA* gene during host cell invasion. Macrophage-derived THP-1 human monocytic cell line was infected with conidia of the wild type, *cpeA* mutant, and complemented strains at MOI 10. After 2-h of incubation, macrophage-infected cells were lysed, and fungus was harvested by centrifugation. The colony forming units (CFUs) isolated from macrophage-infected cells were counted, and the percentage of *T. marneffei* killed by macrophageswas calculated. Each bar depicted the average and SD values obtained from triplicate experiments. **(B)** Stress tolerance was assessed in the Δ*hsp30* mutant. One thousand conidia from the *T. marneffei* wild-type and Δ*hsp30* strains were spotted on the surface of minimal medium containing various stressors, including 1 M sorbitol (osmotic stressor), 0.2 M NaCl (hyper-salinity), and 1 mM hydrogen peroxide, H_2_O_2_ (oxidative stress). The plates were incubated at 37°C for 10 days and the results were photographed. Experiments were performed in three replicates.

To provide proof-of-concept that information from our antigenic-protein profiling could lead to an understanding of the interplay between the host and pathogen interaction, we sought to characterize the functions of these antigenic proteins under physiological conditions. First, we turned our attention to the catalase-peroxidase encoding gene, *cpeA*. The immunogenic property of the CpeA protein was confirmed, and the Δ*cpeA* mutant was previously constructed (Pongpom et al., 2005; Pongpom et al., 2013). The Δ*cpeA* mutant showed dramatic growth reduction when the cells were exposed to hydrogen peroxide, indicating its role in the oxidative stress response (Pongpom et al., 2013). However, the involvement of the CpeA protein in the interaction with host cells during fungal invasion and infection remained unknown. In this study, the human cell line THP-1, differentiated into macrophage-like cells by PMA treatment, was infected with *T. marneffei* conidia at MOI 10 and assessed the percentage of fungal strains killed by the macrophages. As shown in **Figure 13A**, the null mutant Δ*cpeA* was 15.2% more susceptible to macrophage killing than the wild type and complemented strains, suggesting that this protein could be involved in fungal survival during macrophage engulfment.

Second, we focused on the *hsp30* gene as it showed the highest levels of gene upregulation during the pathogenic yeast phase (**Figures 3**, **5C**) and the Hsp30 protein has previously been validated to be immunogenic (Vanittanakom et al., 2009). The *hsp30* gene was subjected to targeted gene deletion, and phenotypic analysis was performed in a null deletion mutant (Δ*hsp30*). We examined the role of *hsp30* during conidiation, germination, and yeast-to-mold phase transition. We found that the Δ*hsp30* mutant exhibited a normal phenotype. Also, the Δ*hsp30* mutant was not sensitive to the anti-fungal drug caspofungin (data not shown). Interestingly, the Δ*hsp30* mutant displayed growth sensitivity to various stressors, and this phenotype was detected only under 37°C-induced yeast morphology (**Figure 13B**). At 37°C, the Δ*hsp30* mutant was sensitive to NaCl (0.2 M), sorbitol (1 M), and hydrogen peroxide (1 mM), suggesting the role of *hsp30* in high salt stress, osmotic stress, and oxidative stress, respectively. Our results demonstrated that *hsp30* functions prominently under elevated temperature, consistent with its highly upregulated levels during the yeast phase where the cells encounter an increased temperature of 37°C.

Together, our phenotypic characterization of the null mutants indicated that both immunogenic proteins, CpeA and Hsp30, contribute to *T. marneffei* stress adaptation under the yeast physiological conditions.

## 4 Discussion

By using sera from infected patients, specific antigens involved with mycotic diseases have been successfully identified in multiple fungal pathogens such as *A. fumigatus* (Shi et al., 2012; Virginio et al., 2014), *C. albicans* (Pitarch et al., 2016), *C. posadasii* (Tarcha et al., 2006a; b), *C gattii* (Martins et al., 2013), *C. neoformans* (Neuville et al., 2000)*, Paracoccidioides* spp. (Moreira et al., 2019), *H. capsulatum* (Almeida et al., 2020), and *S. schenckii* (Rodrigues et al., 2015). This study provided the expanded list of antigenic proteins involved in human and *T. marneffei* interaction. We found some of the same protein families from several fungal species showed the ability to stimulate antibody production. For instance, many heat shock proteins have been detected using patient serum antibodies, including Hsp88, Hsp90, Hsp1, Hsp70, and Hsp60 from *A. fumigatus* (Virginio et al., 2014); Hsp90 and Hsp70 from *C. albicans* (Pitarch et al., 2016), Hsp60 and Hsp70 from *C. posadasii* (Tarcha et al., 2006a); and Sks2 from *C. gatti* (Martins et al., 2013). Consistently, the Hsp30 protein has been shown to function as an immunogen in *T. marneffei* (Vanittanakom et al., 2009). Catalase was identified as an immunogen in *H. capsulatum* (Almeida et al., 2020) while the catalase-peroxidase bifunctional enzyme was also characterized as an immunogen in *T. marneffei* (Pongpom et al., 2005). NADH-ubiquinone oxidoreductase from *H. capsulatum*, *C. posadasii* and *T. marneffei* can elicit a human immune response (**Table 1**, Tarcha et al., 2006a; Almeida et al., 2020). Also, several ribosomal proteins and translation factors exhibit antigenic properties in *H. capsulatum* (Almeida et al., 2020), *Aspergillus fumigatus* (Virginio et al., 2014), *Coccidioides posadasii* (Tarcha et al., 2006a)*, Cryptococcus gatti* (Martins et al., 2013), and *Candida albicans* (Pitarch et al., 2016), which agrees with our finding of the 60S ribosomal protein, RPL20A, being an antigenic protein (**Table 1**). These studies together identify the fungal proteins that are commonly involved in interactions between host and pathogen, leading to an immune response during mycotic infection.

All proteins from our antibody screening experiment were predicted to contain a high percentage of B-cell epitopes, consistent with the role of the identified proteins in stimulating an antibody response. Importantly, all antigenic proteins contain overlapped peptide regions that were simultaneously identified by three different B-cell epitope prediction tools. Indeed, some antigenic proteins, such as P26 (Epitope = 95.7%), Nuo21.3 (Epitope = 81.8%), and Nbr1 (Epitope = 73.7%), were mapped with a remarkably high percentage of B-cell epitopes; over 70% of the entire protein was predicted to be epitope regions. Thus, our antibody screening approach successfully identified proteins with high antigenicity. The immunogenic properties of these proteins (P26, Nuo21.3, and Nbr1) and their epitope regions identified herein need to be investigated in future studies.

To our surprise, most identified antigenic proteins were predicted to have intracellular localization. Indeed, most proteins seem to localize in membranes, membrane-bound organelles and/or cytoplasm. To our knowledge, this is the first study to discover the antigenic properties of the membrane-trafficking proteins TmHam13, TmMon1, TmFus1, TmPho88, and TmNbr1. These proteins have a putative role in membrane trafficking processes, including membrane fusion, signal transduction, and autophagy. Recently, extracellular vesicles (EVs) have been the focus of many studies because they are secreted by various cells from a divergent range of organisms (Rizzo et al., 2020). In pathogenic organisms, EV secretion usually promotes disease through the delivery of simple molecules and virulence-associated factors (Rodrigues et al., 2008; Baltazar et al., 2018; Park et al., 2020). Thus, pathogenic EVs seem to be loaded with immunogens and inflammatory activators that can stimulate the host immune response. Accordingly, we postulated that these intracellular proteins could be accessed by the host immune cells through EV transport during *T. marneffei* infection. Proteomic analysis of EVs from *T. marneffei* has been characterized (Yang et al., 2020). Strikingly, NADH-ubiquinone oxidoreductase, glutathione peroxidase, ribosomal protein L20, heat shock protein 30, and fructose-1,6-bisphosphatase are found in EVs, supporting our speculation. We noted that the study by Yang et al. used a different *T. marneffei* strain from ours, and EVs were isolated from *in vitro* cultures. The contents of EVs can change depending on the microenvironment (Baltazar et al., 2018; Cleare et al., 2020), so it may be possible that other intracellular proteins are packed into EVs, transported and exposed to host immune cells during infections. In addition, we hypothesize that membrane-associated proteins identified here might participate in EV biogenesis, sorting, and secretion to deliver multiple and diverse cargos required in the human-*T. marneffei* interaction. This is an interesting field to explore in the future as more experiments are necessary to prove our hypotheses and fill in this knowledge gap.

In addition to their antigenic nature, some of these novel membrane-associated encoding genes are strongly implicated in fungal virulence. For example, Mon1 plays an essential role in fungal pathogenicity in three plant fungal pathogens, *Fusarium graminearum*, *Magnaporthe oryzae* and *Valsa mali* (Liu et al., 2012; Gao et al., 2013; Li et al., 2015; Xu et al., 2022). Also, Mon1 is required for full virulence in some human fungal pathogens such as *C. neoformans* (Son et al., 2018). In another human fungal pathogen, *C. albicans*, the fatty acid desaturase gene Ole1, the homolog of *T. marneffei* SdeA, plays a critical role in virulence (Xu et al., 2009). Notably, Pho88 shows genetic interaction with Ole1, yet the virulent role of Pho88 has not been verified in the study by Xu et al. It would be interesting to investigate if the genes identified from our screening are implicated in virulence and other virulence-related phenotypes in *T. marneffei*.

As autophagy relies heavily on membrane dynamics to form the membranous structures, factors that play a role in membrane trafficking are essential for autophagy (Yang and Rosenwald, 2014). Our antibody screening experiment identified three membrane-related genes that play an extended role in autophagy, TmMon1, TmNbr1, and TmPho88. To our knowledge, the vacuolar fusion protein Mon1 is required for normal vacuole formation and autophagy in all tested species, including *S. cerevisiae*, *M. oryzae, F. graminearum*, *C. neoformans*, *Aspergillus* species and *V. mali* (Meiling-Wesse et al., 2002; Gao et al., 2013; Li et al., 2015; Son et al., 2018; Son and Park, 2019; Xu et al., 2022). Moreover, Nbr1 is the receptor protein that mediated vacuolar targeting of cargo transports in the autophagy pathway (Mizushima, 2015). The role of Nbr1 in autophagy is strikingly conserved across eukaryotes. In mammals, Nbr1 is a cargo receptor for the degradation of ubiquitinated substrates (Kirkin et al., 2009; Kuo et al., 2011; Deosaran et al., 2013; Kenific et al., 2016). In the plant *Arabidopsis thaliana*, Nbr1 functions in selective autophagy of protein aggregates and viral proteins during the plant stress response (Zhou et al., 2013; Hafrén and Hofius, 2017; Jung et al., 2020). In the filamentous fungus *S. macrospora*, Nbr1 mediates selective pexophagy (Werner et al., 2019). Lastly, Pho88 is a key component of the ER targeting pathway. While the specific function of Pho88 in other fungal species remains to be elucidated, the role of Pho88 in mitophagy and autophagy has been demonstrated in *S. cerevisiae* (Peselj, 2015). Our finding that autophagic proteins could elicit antibody production might imply that autophagy could play an important role during host colonization, invasion, and infection.

Identification of antigenic proteins could guide researchers to further understand the host-fungal pathogen interactions. As exemplified by our current results, gene expression profiling, coupled with targeted gene deletion of the identified *cpeA* and *hsp30* genes, provided information that CpeA and Hsp30 proteins likely aid *T. marneffei* in coping with host-derived stress during macrophage killing and infection. Thus, the complete report on antigenic protein profiling will be beneficial for not only the development of therapeutic and diagnostic interventions but also the knowledge of the interplay between host and pathogen.

Another one of the strengths of our study was that all antigenic proteins identified here could be detected in patients with an immunocompromised state. Thus, the diagnostic value of these antigenic candidates is encouraging from a clinical perspective. However, it has been shown that some of the fungal antigens can trigger antibody production even in healthy people (Pongpom et al., 2013; Almeida et al., 2020). Indeed, the *H. capsulatum* study revealed that only three out of 132 identified antigenic proteins are specifically recognized by antibodies in sera of histoplasmosis patients without a cross-reaction with sera from other health conditions (Almeida et al., 2020). Most fungal pathogens are simply contaminants, and therefore individuals can be exposed to, and produce antibodies against, those fungi without the presence of illness, especially in endemic areas. Thus, these antigens could possibly be used as markers for the detection of previous exposure. Further testing is required to validate whether or not our antigenic candidates are specific proteins that elicit antibody production only in talaromycosis patients.

## 5 Conclusion

Our major finding here was the identification and characterization of *T. marneffei* proteins recognized by antibodies from immunocompromised patients with talaromycosis. The applied bioinformatic methods allowed us to analyze putative functional domains, critical residues, cellular sorting signals, protein localizations, and epitope regions. Notably, the identified antigenic proteins possess functional domains related to membrane trafficking, autophagy, and other crucial cellular processes. These proteins, including TmMon1, TmFus1, TmHam13, TmNbr1, TmPho88, TmMarvel, and P26 showed specific gene expression profiles in different growth phases, suggesting their importance in distinct physiological contexts. Further analysis of selected antigenic proteins provides evidence for their possible roles in intracellular survival. The CpeA protein played a crucial role in the oxidative stress response and fungal survival during macrophage engulfment. Besides, Hsp30 was implicated in stress adaptation, particularly under elevated temperatures, highlighting its significance during the yeast phase. Overall, our study emphasizes antigenic protein profiling in unraveling the complex mechanisms underlying fungal infection, ultimately contributing to the development of improved diagnostic tools, therapeutic interventions, and prevention measures against *T. marneffei* infections.

## Data availability statement

The DNA sequences of antigenic protein-encoding clones in the study are deposited in the Genbank database, accession number: P1; OQ241945, P3; OQ241946, P6; OQ241948, P7; OQ241949, P9; OQ241950, P10; OQ241951, P11; OQ241944, P12; OQ241952, P13; OQ241953, P14; OQ241954, P15; OQ241955, P17; OQ241956, P21; OQ241957, P23; OQ241947, P24; OQ241958, P26; OQ241959, P28; OQ241960. Further inquiries can be directed to the corresponding author.

## Ethics statement

We obtained the human sera as anonymous blood samples from the laboratory unit (blood biobank). All patients consented to treatment at Maharaj Nakorn Chiang Mai Hospital. The consent requirement and research protocol complied with this research fall into the exemption category according to the announcement from the Research Ethics Committee of the Faculty of Medicine, Chiang Mai University.

## Author contributions

MP performed the experiments and collected data. MP and TW analysed and interpreted data. TW and MP participated in writing and editing the manuscript. MP, TW, and AA generated figures and tables. All authors contributed to the article and approved the submitted version.

## Supplementary material

Supplementary Figure 1 The Tm Mon1 interaction network shows enrichment in the vacuolar trafficking process. Predicted partners of Tm Mon1 are related to vacuolar protein sorting-associated protein (Vps proteins), Rab small GTPases, Rab GDP dissociation inhibitor (GDI), and vacuolar fusion protein Ccz1. Tm Mon1 was subjected to STRING analysis. Full protein IDs are as follows: Rab7 = XP_002144848.1; Ccz1 = XP_002144339.1; Pep3_Vps18 = XP_002145427.1; Vps11 = XP_002145309.1; Fructose-2,6-bisphosphatase_Vps39 = XP_002148716.1; Sec1_Vps33 = XP_002153114.1; Glutamate decarboxylase = XP_002143987.1; Rab GDI = XP_002152034.1; Vps9 = XP_002151504.1; Vps8 = XP_002150156.1; Vps16 = XP_002145095.1; Vps39_2 = XP_002146004.1.

Supplementary Figure 2 Protein sequence alignment of hyphal anastomosis Ham-13 is depicted. Ham-13 protein contains two fungal conserved domains with unknown functions, DUF4449 **(A)** and DUF5923 **(B)**. Protein sequence alignment was performed using Ham-13 and homologous proteins from the following species: SPAC32A11.02c = *S. pombe* (NP_593775.1); Nc = *Neurospora crassa* (NCU06265, XP_962869.1) Tm = *T. marneffei* (XP_002149306.1). Shaded green highlights the DUF4449 domain **(A)** while shaded cyan highlights the DUF5923 domain **(B)** identified in Ham-13 protein sequences from *T. marneffei*.

Supplementary Figure 3 The Tm Nbr1 interaction network shows enrichment in the autophagy-related process. Predicted partners of Tm Nbr1 are related to autophagy-related proteins (Apg and Atg proteins) and ubiquitination pathways. Tm Nbr1 was subjected to STRING analysis. Full protein IDs are as follows: Atg8 = XP_002149194.1; ubiquitin-60S ribosomal protein L40 fusion protein = XP_002150296.1; Polyubiquitin = XP_002151173.1; ubiquitin fusion degradation protein C12B10.01c = XP_002146650.1; STIP1 likey and U box-containing protein 1 = XP_002152020.1; Apg5 = XP_002150442.1; Atg7 = XP_002144542.1; Atg12 = XP_002144707.1; Ubiquitin domain-containing protein DSK2 = XP_002145807.1; Apg9 = XP_002147500.1.
