# Supplementary figures and images for: Antibody screening reveals antigenic proteins involved in Talaromyces marneffei and human interaction
Source: Front Cell Infect Microbiol. 2023 Jun 19;13:1118979. doi: 10.3389/fcimb.2023.1118979 (PMC10315666; doi:10.3389/fcimb.2023.1118979)

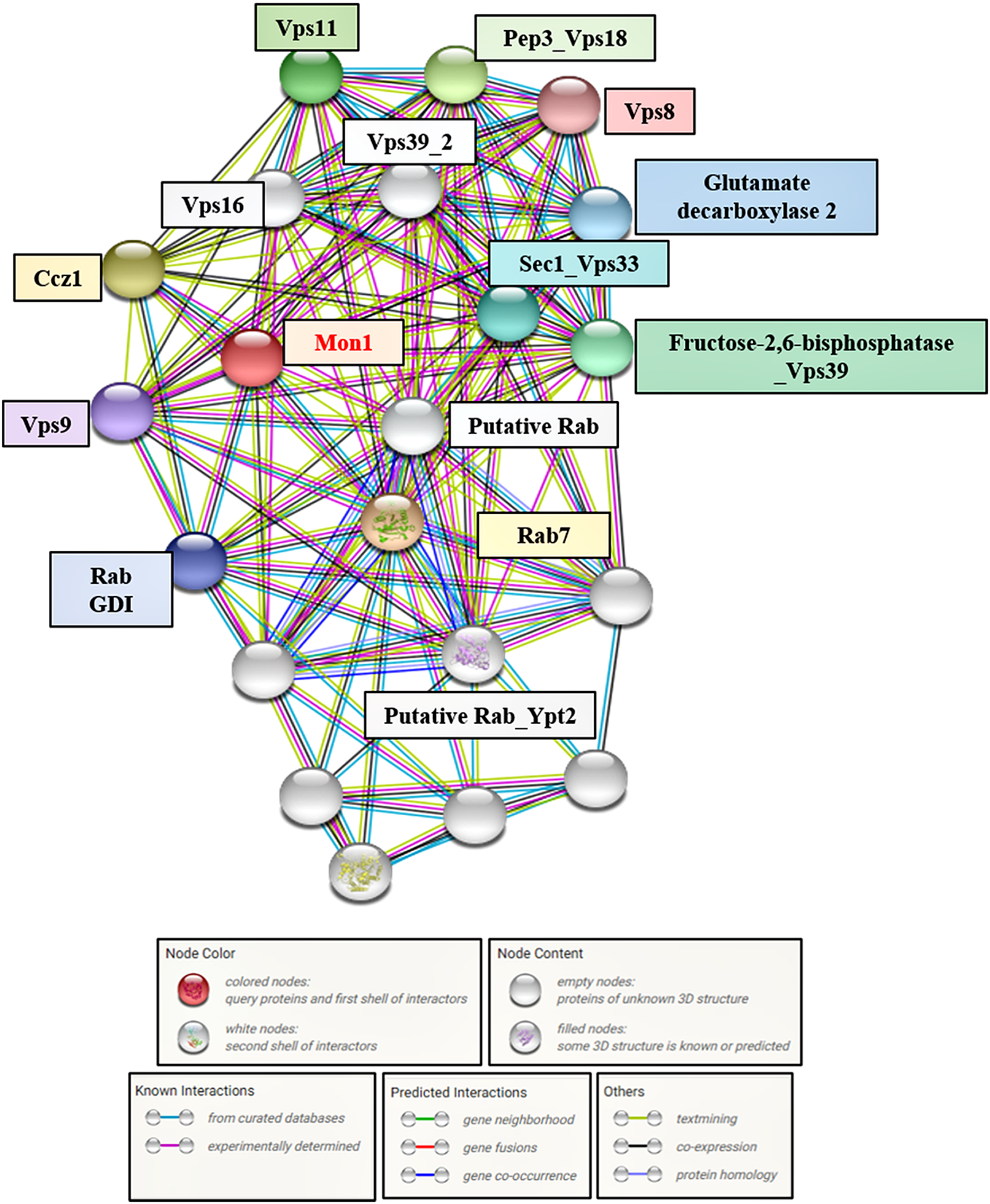

Supplement: Supplementary Figure 1 — The Tm Mon1 interaction network shows enrichment in the vacuolar trafficking process. Predicted partners of Tm Mon1 are related to vacuolar protein sorting-associated protein (Vps proteins), Rab small GTPases, Rab GDP dissociation inhibitor (GDI), and vacuolar fusion protein Ccz1. Tm Mon1 was subjected to STRING analysis. Full protein IDs are as follows: Rab7 = XP_002144848.1; Ccz1 = XP_002144339.1; Pep3_Vps18 = XP_002145427.1; Vps11 = XP_002145309.1; Fructose-2,6-bisphosphatase_Vps39 = XP_002148716.1; Sec1_Vps33 = XP_002153114.1; Glutamate decarboxylase = XP_002143987.1; Rab GDI = XP_002152034.1; Vps9 = XP_002151504.1; Vps8 = XP_002150156.1; Vps16 = XP_002145095.1; Vps39_2 = XP_002146004.1. [file Image_1.tif]

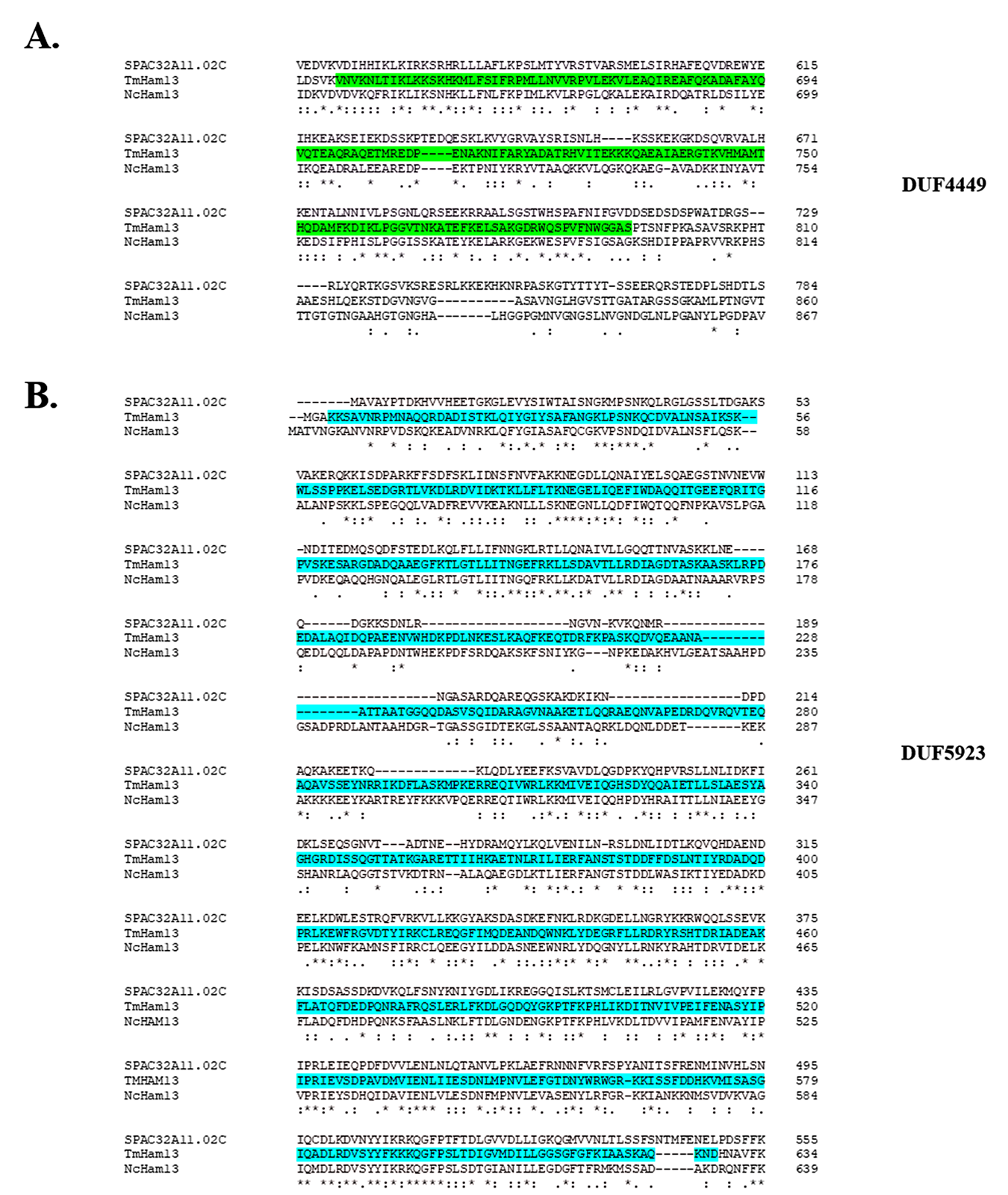

Supplement: Supplementary Figure 2 — Protein sequence alignment of hyphal anastomosis Ham-13 is depicted. Ham-13 protein contains two fungal conserved domains with unknown functions, DUF4449 (A) and DUF5923 (B). Protein sequence alignment was performed using Ham-13 and homologous proteins from the following species: SPAC32A11.02c = S. pombe (NP_593775.1); Nc = Neurospora crassa (NCU06265, XP_962869.1) Tm = T. marneffei (XP_002149306.1). Shaded green highlights the DUF4449 domain (A) while shaded cyan highlights the DUF5923 domain (B) identified in Ham-13 protein sequences from T. marneffei. [file Image_2.tif]

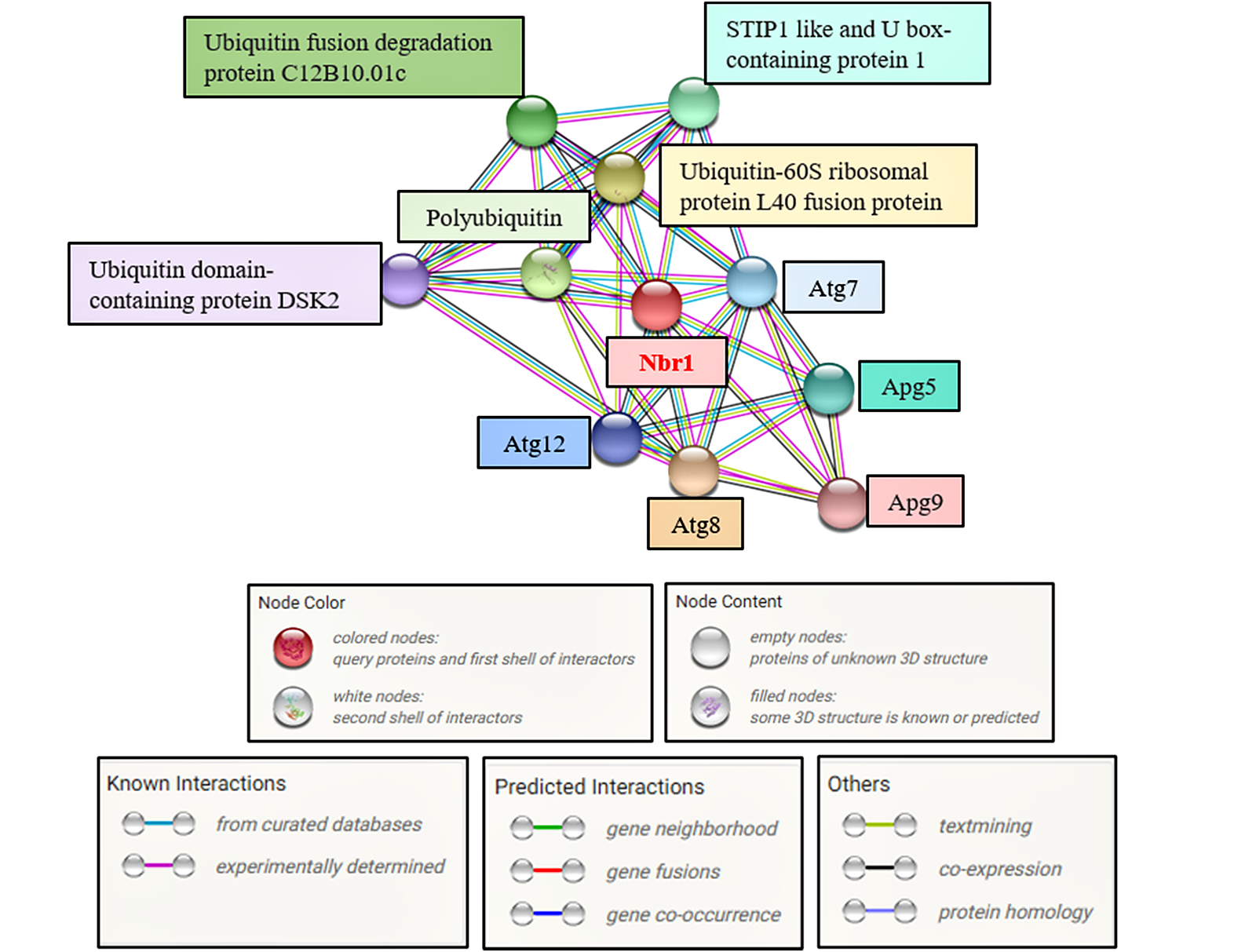

Supplement: Supplementary Figure 3 — The Tm Nbr1 interaction network shows enrichment in the autophagy-related process. Predicted partners of Tm Nbr1 are related to autophagy-related proteins (Apg and Atg proteins) and ubiquitination pathways. Tm Nbr1 was subjected to STRING analysis. Full protein IDs are as follows: Atg8 = XP_002149194.1; ubiquitin-60S ribosomal protein L40 fusion protein = XP_002150296.1; Polyubiquitin = XP_002151173.1; ubiquitin fusion degradation protein C12B10.01c = XP_002146650.1; STIP1 likey and U box-containing protein 1 = XP_002152020.1; Apg5 = XP_002150442.1; Atg7 = XP_002144542.1; Atg12 = XP_002144707.1; Ubiquitin domain-containing protein DSK2 = XP_002145807.1; Apg9 = XP_002147500.1. [file Image_3.tif]
