# Supplementary material for: Antibody screening reveals antigenic proteins involved in Talaromyces marneffei and human interaction
Source: Front Cell Infect Microbiol. 2023 Jun 19;13:1118979. doi: 10.3389/fcimb.2023.1118979 (PMC10315666; doi:10.3389/fcimb.2023.1118979)
Supplement: Supplementary file 4 [file DataSheet_1.pdf]

Fasta format of P clones

>P1

ATGGCTGAGAGCAAGTGTCCCGCTCACCAGCATGTGTTGAAGGCCAACGTCGGCGGTGCTGGTACCAGC
AACCAAGATTGGTGGCCAGACCGCTTGAAGCTTAACATCCTCCGCCAAAACAACCCCGTCTCCAACCCT
CTGGGCGAGGAATTTGACTATGCCGCCGCCTTCAACAGCCTAGATTACTTTGCGCTCAAGAAGGATATT
CAAGATCTGATGACTGACTCCCAGGACTGGTGGCCGGCTGACTTTGGCCACTATGGTGGTCTCTTTATT
CGTATGGCCTGGCATAGTGCCGGTACCTACCGAGTCGCCGACGGTCGAGGCGGCGGTGGCGGCGGCCAA
CAGCGCTTTGCTCCTCTCAACAGCTGGCCCGACAATGTCGGTCTCGACAAGGCCCGCCGTTTGTTGTGG
CCCATCAAGCAGAAATACGGAAACAAGATCTCGTGGGCGGATCTCCTATTGCTCACTGGTAACGTCGCC
CTTGAGTCCATGGGTTTCAAGACCTTTGGTTTCTCTGGCGGTCGTGCCGACACATGGGAAGTGGATGAG
TCAGCCAACTGGGGAGGGGAAACCACCTGGCTAGGCAATGACGTCCGCTACTCCGGCGGTAAGGCTGAT
CACAAGGATATCCACAACCGTGACTTGGACAAGCCACTGGCCGCTGCCCACATGGGTTTGATCTATGTC
AACCCCGAAGGTCCTGATGGAAACCCCGACCCCATCGCCGCTGCCAAAGATATTCGCACCACCTTCGGT
CGTATGGCCATGAACGACGAGGAGACGGTTGCCCTTATTGCCGGCGGTCACACCTTCGGTAAGACACAC
GGTGCTGGCCCAGCAGACAAGCTCGGCCCGGAACCAGAGGCTGCAGACATGGCACAACAGGGTTTAGGC
TGGACCAATAGCTTCAAAAGCGGCAAGGGTCCTGATACCACAACCAGCGGTCTCGAAGTTACCTGGACC
AAGACTCCTACTAAATGGAGTAACCAATTCTTGGAGTACCTCTTCCGCTACGACTGGGAACTCACTAAG
AGTCCTGCCGGCGCCCACCAGTGGGTCGCCAAAAATGCAGAGGCTTTCATCCCCGATGCATTTGACCCA
TCCAAGAAGCGCAAGCCAATGATGCTCACGACCGATCTTTCCCTTCGCTATGACCCTATCTACGAGAAG
ATCTCTCGTCGCTTCTTGGAGCACCCTGACCAGTTTGCTGATGCGTTTGCCCGTGCCTGGTTCAAGTTA
CTTCACCGTGACCTTGGCCCACGAGCTCTCTACATTGGTCCCGAAGTGCCTGCAGAGGTTCTACCCTGG
CAGGATCCCGTTCCCGCTGTCGACCACCCCCTCATTAGCAATGAAGACGCGTCGGCTTTGAAACAGCGC
ATTTTGGCCTCGGGTGTCAAACCATCCAGCTTGATTTCCACTGCTTGGGCATCCGCTTCTACGTTCAGA
GGTAGCGACAAGCGCGGCGGTGCCAATGGTGCTCGCATCCGCCTGTCTCCTCAGCGTGAGTGGGCAGTT
AACAACCAACCCTGGTTGCGCGAGACCCTTTCTGTGCTTGAAGCCATACAGAAACAATTCAATACCTCC
CAGTCTGGAGGCAAGAAGGTGTCTATTGCAGACTTGATTGTTCTCGCTGGTGTCGCCGCTGTTGAGAAG
GCTGCTCGCGACGCCGGATACGCCGTCACAGTACCCTTCACTCCCGGTCGCACAGATGCTTCCCAAGAG
CAGACTGACGTCCAATCCTTCAGCGACATGGAGCCCATTGCTGATGGTTTCCGTAACTACGGCTCATCC
ACCTCTCGCGTTCGTGCTGAGGAGTGGCTCATCGATAAGGCACAGCTTTTGACCCTCAGTGCACCCGAG
TTGGCCGTTCTCATCGGCGGTCTCCGTGTCCTCAACACAAACTACGACGGCTCTGCTCACGGTGTCTTC
ACCCAGCGCCCAGGCAAGTTGACCAACGACTTCTTCGTCAACCTCTTGGACATGAACACCGCATGGAAA
TCAATTGGTGGTGTCGACCTCTACGAGGGCACAGATCGCAAGACTGGCGCCAAGAAGTGGACTGCTACT
CGTAACGATCTCGTCTTTGGCTCCAACGCTGAGTTGCGTGCTATTGCTGAGGTGTACGGTAGCTCTGAT
GGCCAGGAGAAGTTCGTCAAGGACTTTGTGGCTGCTTGGGACAAGGTCATGAACTTGGATCGATTCGAC
TTGAAGAAGAAGCAATCCACTTCCAGTCACCGCCTTTAA

>P3
ATGTTCAAGAAGCAGCTAATGCTGCTACTACTGCTGCAACTGGTGGTCAGCAAGATGCTTCAGTCTCTC
AGATTGACGCACGTGCTGGTGTCAATGCTGCCAAGGGAGACCCTGCAACAGCGCGCCGAGCAGAATGTT
GCACCTGAAGACCGGGACCAAGTTCGACAGGTTACTGAACAAGCACAAGCGGTATCTTCTGAATATAAT
CGGCGTATTAAGGATTTCTTGGCCTCCAAGATGCCCAAAGAACGTCGTGAACAGATCGTTTGGCGTTTG
AAGAAGATGATTGTTGAGATCCAGGGTCACTCTGATTACCAACAAGCCATCGAGACTCTCTTGAGTTTG
GCTGAGTCATATGCTGGACATGGAAGAGATATCTCTTCGCAAGGAACGACCGCTACTAAAGGGTTCATG
GATAGCAAGAAGGACATGCTGATGCGACTCAAGATTTTAATCGAGAGATTTGCGAATAGCACATCGACC
GATGACTTCTTTGATTCGCTCAACACGATCTATCGCGATGCCGACCAGGACCCGCGGTTGAAGGAATGG
TTTAGGGGTGTCGACACTTACATTCGAAAATGCCTGCGCGAGCAAGGGTTCATCATGCAAGATGAAGCT
AATGACCAGTGGAACAAGCTGTACGATGAGGGTCGCTTCTTGCTTCGAGACCGGTACAGAAGTCACACA
GATCGTATTGCCGATGAAGCCAAGTTTTTGGCCACTCAATTTGACGAAGATCCACAGAACCGAGCTTTC
CGTCAATCTTTGGAGAGACTCTTCAAGGATCTGGGTCAGGACCAGTATGGAAAGCCGACGTTCAAGCCC
CATTTGATCAAAGACATTACCAATGTCATCGTTCCTGAGATATTTGAGAATGCCAGCTACATTCCTATT
CCTCGAATTGAGGTTTCCGACCCAGCTGTTGACATGGTTATCGAAAACCTTATCATCGAGAGCGACAAC
TTGATGCCCAATGTTTTAGAATTCGGCACTGACAACTACTGGCGGTGGGGTCGTAAGAAGATCAGCAGC
TTCGACGACCACAAGGTTATGATTTCTGCATCTGGCATCCAAGCTGATCTCCGTGATGTGAGCTACTAT
TTCAAGAAGAAGCAAGGATTCCCTTCCCTCACCGACATCGGTGTCATGGACATTCTTCTTGGTGGTTCA
GGCTTCGGCTTCAAGATTGCTGCCTCCAAAGCACAGAAGAATGACCACAACGCAGTTTTTAAGCTTGAT
AGTGTCAAGGTCAACGTGAAGAACCTTACCATCAAGCTGAAGAAGTCCAAGCACAAGATGCTTTTCATC

ATCTTCCGACCCATGCTGTTAAATGTTGTCCGACCTGTTCTTGAAAAGTGTTGGAGGCCCACATTCCTG
AGGCATTCCAAAAGGCAGACGCATTTGCCTACCAGGTACAGACCGAGGCTCAGCGTGCCCAGGAGACCA
TGCGTGAGGATCCTGAAAATGCAAAGAATATCTTCGCCCGCTACGCCGATGCTACTCGTCACGTCATCA
CTGGAGAAGAAGAAGCAGGCCGAAGCTATCGCCGAGCGCGGAACCAAGGTTCATATGGCAATGACTCAC
CAGGATGCCATGTTCAAGGACATCAAATTGCCTGGTGGTGTCACTAACAAAGCTACTGAGTTCAAGGAG
CTTTCTGCTAAGGGTGATCGCTGGCAATCACCAGTTTTCAACTGGGGCGGTGCTTCACCCACTAGCAAC
TTTCCCAAGGCTTCCGCAGTGTCTCGCAAGCCTCATACTGCCGCTGAAAGTCATCTGCAGGAGAAGTCA
ACCGATGGTGTCAATGGAGTCGGAGCTAGTGCAGTCAATGGACTCCACGGAGTCTCGACGACTGGAGCC
ACAGCTAGGGGATCCAGTGGCAAGGCAATGTTGCCAACCAATGGTGTCACCAACGGCCAGTCTGCCACC
AACGGTACTTTCCAGAAGGAAGTCGAGCGTGCTTTTGACGCAAACGGCACCACTCTTCCAACCCTAGGT
GGTGTTTAA

>P23
ATGTCTCTCTTCCACCGCAGCGGCGACTTTGCTCCCCTCTTCCGTCTCCTCGACGACTATGACCTCCAC
CGCTCCGGACGCGACGGTCAGACTCCTGCCTCCAGTAGCATCTCAAGCTTCGCGCCACGCTTCGACGTC
CGCGAGTCCAAGGATGCTTACCATCTGGACGGCGAACTCCCCGGCATTGCTCAAAAGGACGTTGAAATC
GAATTCTCCGACCCGCAGACATTGACCATCAAGGGTCGCTCGGTTCGGGAATACCACACCCTTCCCGAG
AACGAGAACCCTCATGCTCCTAAGCCCGCTTCTGTCGAAGACGCACCCGAGTCCAGTGACGAGACAGCC
GTCCAAAAGTCTTCCGACAAAAAAGAGGTCTCGAAGGCTCAGGGTAACGGCTACAAGTACTGGGTCAGC
GAGCGCTCAGTCGGCGAGTTCCATCGCTCATTCAACTTCCCTAGCCGCGTTGATCACAATGGCGTCAAG
GCTAGTTTGAAGAATGGTGTTCTTACGGTGACGGTGCCCAAGGCTGCCCCTCCTACTAGTCGCAAGATC
ACAATTGAGTAA

>P6
ATGGAGACTGCGAACGACGCACCCTCAACTACACTGGAGGGACAACATGAAGCAGACGCTCAGAACAGC
CGAACTCAACAACCCCAACAATCCCCCGACAGTCAAGAAGACGACGCAGAAGAAGTACGACCACCATTG
CCACCACGTCCTGAGACCATCGATCTGCTAAATGAAGGCATTGCCTTTCGTACCTCCACGGCGAGACCA
AACCTGCAGTCGCACGCGACAACGGCACTGTCATTGACAGACATTACCGGCCAAACGAATGCAGATGGA
AGAGACGGTTTCGTTGCAGGCTTTGGGCGTACCTTGCTGGGGCGGGGACTACGAGCAAAGGCTAGTTTG
AGCCAACTGAATAGTGCTCGTGGTAGTGAAGCCGGGGACACCGCGAGCGTGTTAAGCTTTGCGCCGAAT
TCCGAGGAAGGCCAGGATGAGAGTCTGTTTGGGGAGTTTGCAAATGAAACCAACGCGCAGGATATTTCT
GGGAATATTGAAGTCCTGGGCTATGATGAATATCCCCAGGACGGCAATGAGTACGAATTTGTAGAGGAA
TTCGAGCCGATAGGGGAACTGGATGAGGATGGCCAAAATGAAGAATCACTGCTCCAGAAATGGAAGGAA
AAGCGAAAGCATTACTTGATTTTATCAGCTGCTGGAAAGCCAATATATACTCGACACGGCGATAGTGGT
TTGGTTTCGGGCTACATTGGTATCATACAAACAATCATCTCATTCTATCAGGACGCAGATGACACTTTG
CGGAGTTTCAGTGCAGGAGATACGAAGATTGTGATACTCTCAAAGACTCCGCTATATTTGGTTGCTATC
AGTCGATTATTGGAAAGCGAAAGCCATCTGAGACTTCAACTTGATGCGCTGTACATGCAGATATTATCG
ACCTTGACCCTTCCGGCATTGAACCATCTATTTTCGATCCGGCCATCTACAGATCTAAAAAGACCCCTA
CAAGGTACCGAGACGTTACTTTCCTCGCTCGCCGATTCTTTTACCAAGGGCTCTCCCACGACTCTCTTA
TCGGCATTGGAGTGCTTGAAACTACGCAAAGCACATCGACAAGTCATCAACAATATCTTACTAAGAAAC
AGAGCGG

>P7
ATGTCGCACGCCCATTTCCACCGTCATGAAAGACGGAATTTCATTTCGGACGTGGAGGATTTCTTTGGG
GTCAATTACGCAAAGGGTCCACAGAGTACTGTAACTGAAACTGCGTCGATTGTATACGTGACTGCGTCG
CCAACCTTCACCGGCCCAATCGGTGGTTATATCACGGGCACAGATACTGCCAGCCCTGCGGATCCAACT
ACAACCGCAGGAAAGGGTGCTCCTGTAGCTCAGTCATCAACAAAGACAACCGCCGCGAGTTCAAAATCA
GACACATCTACCACCACTAAGGTTCCTGCTCCCACGACTCATACTACTTCAACTCTCTCGACAACGTCA
ACCTCGCTGTCCGACTTGTTGACCACCTCGAGCGATTCTCCTGCGACAACTTTCTTGACGTCTTCTTCA
ACGCAATCTGCATCTTCCACTTCAACCGATACAGCACTAGACCAGCTCTCGTCTTCTCCCACTGCTTCT
CCCACTACTTCTGCAGCCGCATCCACTTCCAATGGTCTTACAGGAGGTGCCAAAGCAGGAATCGCCATT
GGTGTCTTGTTCGGCGTTGGTCTTATTGCTGGATTGATTCTGTTCTGGCTACATAAGCAGAAGAAGAAC
AGAGAAGAAGCGGCTGCTGCTGCCGCTGCTGCAGAAAATGAGAAATTTACTCCAAGTCAACCCCCACCA
ATGACTAGCGCTCAGTCACCACAGTCAATGGCAGCTTACTCTTCTGCGCCGTCAACTCCAGCGACTGCC
CCTCAAGTCAGCTTGCGTCCGATCACTCAGTTCAACCCTTTGTTGAGCCAGCCTGGTGGTGCCAATCCT
TACGCCGCTGGTGCTGTAGGTGCTGCCGTCGGTGGTGCTGCTGCTGCTGCCGGTGGCCTACAAGTAAAT
CGGTCAGCTGAACGCCCTTACAGCGGCTCAGCACATGTTCCTCCTCAGTCTCCTCGTCAAGATCCATTC
ACTGATCCTGTTAATCCTTTCGATAATGGAGCGCAAACTGCATCTCCTCCTATGCCCCCTGCTAAAGAT

GCATCGTCTCCAGTCAGGGATTTGACGCCTTCGCCTACCGGTAGTGCCCACAACTTGGCCAGCCCTATT
GCTGAAGAACCGTCCGCTGGGTCAGTAGAAGCTGCCGCTGCCGGTGCTGTCGCAGGTGCTGCTGTTGGC
GCTGCTGCTGTTGC

>P9

CGTTGTTCGTGTAACGAATACCTCCATACCTATACTTGTATATCCGACGTACGTACAGCTACGGCTCTT
CTCCGCTGAACCGAGTTCCTCACATCGTCGATATTCTTGTATCTCAGCAAAAATAGATCACCAATATCC
TAAACGATCATCACCACAATGGCAGCCAACGGCAATGGCGGTTCCGGCGTTGGCCAGGAGAACATCAAC
ACAGATATCATCACTCTCACACGATTCCTGACGGAAGAGCAGCTTCGAGTCCCAGAAGCTACCGGTGAT
TTCACTCTCCTCTGCCACGCCCTGCAATTCGCTTTCAAGTAATTGCCTACTACATTCGACGAGCCAGCC
TCATCAACCTCACCGGTCTAGCCGGCTCCTCCAACATAACCGGCGACGACCAAAAGAAACTCGACGTAA
TCGGCAACGATGTCTTCATCGCCGCCATGCGCGGCTCAGGCAAGGTGCGCCTGCTCGTCTCCGAAGAAG
AAGAAGAGGCAATCATCTTCG

>P10 (436)

AAACGACACAAAATGGGCCGTCTCAACGAGTACCAGGTCATCGGGCGCCACTTGCCCACCGAGGCAAAC
CCGACCCCCAAGTTGTACCGCATGCGCATCTTTGCTCCCAACACAGTTGTTGCCAAGTCGCGCTTCTGG
TACTTCCTCACCAAGCTCAAGAAGGTCAAGAAGGCCAACGGTGAGATCGTCAGCCTCAACGTGATCTCC
GAGAAGCGTCCCACCAAGGTCAAGAACTTCGGTATCTGGTTGCGTTACGACTCTCGCTCCGGCACCCAC
AACATGTACAAGGAGTTCCGTGAGCTCAGCCGAACTGATGCCGTCGACTCTTTGTACCAGGACATGGCT
GCCCGTCACCGTGCCCGCTTCGGCTCCATTCACATTCTCCGCGTCATCGAGATCGAGGACAACGAGAGC
ATCCGCCGCCCTTACATCAAGC

>P11 (433)
GACTCCTCAAAAGAAATGGACGTAGTTTCAAACGAAAGACTGGACGTGAGTTGATCTCGTTGACTCCCA
AACTTAACGGAAAGACAAAATGTTCTTCGAGGAAGGAGGACACTTTGCCCGTTGCAGGAAAGCAGTCAC
GTGAGCCCACTATCAGTGGCAACGGCGGAGCACCGCGGACAGAGCGAACCAATAAACAGCCGCAAACCG
GGGAAGCTAATCTAATCCATAGAACGATTTGGAAGCTCTACGATGGCGCCACAGATACCAGCAAACTAG
TCATTTTGATACATAACAGAATCCGTGGTATGAGTGAATGGTCTTAGCAGCTGAATAAATCCACAATAT
AGCCAGCCCATAACGAGTCAGCAACTAGTAGTATATGGCGGATAAGATAGAGCTTCCCCCACATTTCAG
CCACGGCTTGTGCACGTCA

>P12 (440)
ATGGCCATGCTCTCGTCATCATTGTCCCGGCCCATCCTCCTGGGCATCCGAACCATGCAATGGGCCAGC
TCCGTCATCGCCTTGGGTATATACGCCTACTTTGTGCATCACCAGCGCAGTGGCACAAACCCCATCTTC
AATCTAGTCATTTCTGTCCTGTCCGTTGTCTTCTTCATCCCTGCTTTCGTGTCGCCGTTCATGACCGTG
CTCAGCAAGTGGGTTGCTTTGATTGATATGGTGTTTTCGTACCTATGGTTGACGGCATTCGTCCTCGCC
GCACAGAGCTACAACTACGGTGATGTCTACTTGAAGGCCCCCTCCGGCC

>P13 (441)
GACCTCACAGTCAGCAGACTCAAAATGTCTCGAGAAGTGGCGAAAGCGGCCAAGTCCGCCTCGAACGCC
ATCGCTGTTTCCAAGAAATACACCGTTCAGTCTACAGGCATCTGGGAAGTGATCCGACGCACACTGGCC
GTCGACCCAACCCGATCCACCGGTGTCCCATTGAACTCTCAATTCCGCAATCCAGCACCAGGCGCCCTA
CCACCTCAATCATACGATGAACCCGTCACTCTCCCCGCCGCCGACCTCGCAGATAACCCTTACTGGAAA
CGCGATGTCCGCCGCAAATACCCTCAACTCAGCGTTTTCAGCCAAGGCGACGTTGCCGGACTTTTGACC
TTTGGAAATAAGCAGGCACCCAAGGAAGACGCTCTTCAGCTTGGAGAGGCTGGTGAGAAGCAATTGATT
GCTGCTAAGCAGGAAGGAGATGAAAAA

>p14 z512)

ACGCCTCACGGTGTACGTGATTCAATGGTTGGCACACCGACCACTGGTCAGAGGACCGGTCGTCGCGGT
CGGCCGCCTGGCGCCAAAAACAAAAACCCTACGAAAGCTACCTTGAAAGCTCTTGTCAAGACTACCGCT
GGGAGCTCTACGAGAGACGCGCAACCCGCCGTACCTGCACCTGCACCTGCTACCTCCGTCCCTGAGCTT
TCCTATCCTATGTTCACGTGTGAATGGGCGTCTTGTCCTGCGCAACTCCACGACGTGCATACGTTGGAG
CGCCATGTTGTCAAAAACCACATATCTGGTCAAACAACATGTCTATGGCAGAACTGTCCCAATCTTGCA

ACAGAGTATAGCGGCGAAGGTTTGAAGGAGCATTTAGCACAAGCGCATATCCAACCTCTGGCATGGAAG
TACGGAGATGGGGCCTCCGTTAATGGAAATGGTGAGAAAGAGATCACCTCACAAGGTTGCTGCTATTGT
GTCATGAATAAATTATTGAGGAAGAACTC

>P15 (485)
TGAAGTTCTACCCTCTCTCGTCGTCCTCGGTCTTTCTACCCAGGCTCTTGCGAGCTCTTACGTCGATTA
TGTTACTAAGGACCAGCATGGTCTTACTGTTTATGAGATGGGTCATAAATATCATTAACACCACTACCT
CAGACTTCAATACCCGGATTCAGAGTTACCAGGTGGTGATCTCAGCAGCATTCTCGAAGGCTGTAATCA
AGTTACCCAAATAATGAAGCTTGGGCTACGATACTCGATCAGCAACAACGAAACCCTTACCAATAATGA
GTGGCTTAGCCTCTCTCACATATGGAGAAAGGGTGGTTTAGAAGATATGTTGAAATGGCTATAAACTAC
TCTTATCCTGAAGAAGTCACTATTCTTGATTCTGGTTGGTTCTAAGCTGGTTAGCGCTTACAGTCAGCA
GATGGCTTCTATAGCCTCGGTGCTAAGTTCTTGAGAGACCCCCAGGAAGGTCGAAGTTCAGAGACACTG
GT

>P17 (332)
ATCCAACTCAATCCGACAGGATTTCGTTCTTACGCTATTCGCATCATCCTAGGCTGTTGTTCGTGACGA
CCCCTCATTTGTATACACAACCGCCTCAGATTAAATCACAACAGCACAATGGCCTCCGCGACCACGTTC
TACGACTTTTCTCCTCCTGACAAAAAAGGAAACCCTTACCCCTTGACAGACTACAAGGGCAAAGTCGTC
CTCGTCGTCAACACAGCATCCAAATGCGGCTTCACGCCCCAATTCGCCGGCCTTGAAAAACTCTACAAA
TCTATCGAAGCCAAGCATCCCGGCGCCTTCACCATCCTGGGTTTTCCCTGCAATCA

>P21 (404)
TCTCCCTCACCACCACCACACAGTATCTTTGTTTTATCATCTTCATCCATCGGTTTTGACCCAGAATAA
TTCTGTCATTTGATTTCCCATCAAAATTCCCGTCGTCATTCATCGAAATCCATAATTTTTCTGGCCCCC
AGCATTCTCCCGTCAGCCTAAGCTTGGTTGAGCATTAGCAGTGCCAAATCAGAACCACTAACTATCATC
GATTTTTCATCACACTGAAAAAAAACTACATTGATCACAACGCCTGATTCAAAATGAGTGATCTGGTGC
CTTTATCAAAATCGTCTGCGATTTTGTCGACCTCCATGGCGACTCCCTCCCCCTCAGCACCGGCCGTCG
GTCCAGACACATTGATCACTATCAAGATTCTTCACAATGACTCGGTTAACCGTCGCTTC

>P24 (440)
ACACGCTAATATTTACAAGGCACGCAACCCCGTACCACGCACCTCTTTATCTTGCTCAAAGCAAGGGTT
ACTTCAAGGATGAGGGACTTAAAGTTGCCTTGCTAGAACCTAATGACCCTTCGGATGTGACCGAGATCA
TTGGCAGCGGCAAGGTTGACATGGGTTTCAAAGCCATGATCCACACGTTGGCTGCCAAAGCCCGTAACT
TCCCGGTTACCTCCTTCGGTTCCCTTCTGGACGAGCCGTTTACTGGAGTTGTCTACCTCAAGGACAGCG
GTATTACCACCGACTTCAAGACCCTCAAGGGCAAACGTATTGGTTACGTTGGAGAGTTTGGCAAAATCC
AAATCGATGAGCTTACCAAGTACTACGGTATGACTCCCGATGACTACACTGCTGTGCGCTGCGGCATGA
ACGTCACCAAGGCCATTATCGAGGGG

>P26 (444)
CTACTGGGCTGAACGCAGACACTATGGCACCGGCATCGGCACCGGCACTCAACCTCATCCACCGCCACA
GACCAGTACCCTCACCACTACCTCCATGCCTCCTGCTAGCACCACCACTACCACCACCAGCGTAAGTGA
ATGGAGCACCGTAACCGAGACGGAGACGAACACAAAGACAGTTTTTGTCCCTTGCTCGACTTCTGTGGG
GACACGTGGGTCGTCGACTGTCTACTCAACTTGGCTTACGACTACTACCTCCGTGTGGACTACGACTCA
TACTACCACCGTTCCTGTCTACGGCACCACGACGACACCTGTTGGTGGTGTTGGTCCCGTTGGATCTGA
GACGGGGCTCGCGTGTCCCTTGCCGGTTACGACTACTCATACGGAGACTAAGACTACTACTGCAACTGT
GACTGTGACTGTGGCCGTGACGGCGCCTGG

>P28 (466)
CACTTTGTCATTGTCATCCATTGTGTGTGTATCTCTGTGACTGACGACTGACAACCAACCACAGCAGCG
CCATGTCTGCTACCACTGCAGATGCCAGGCCTCGGCCTGCTGCGGACACCAAGAAGGTGCACATCGCCG
ACACAAAAATGACCCTTAAGAATTGGTATAAGCATGTCGACTGGTTGAATGTGTACTTCATCATCGGTA
TTCCACTCTATGGATGCATCCAGTCACTCTGGGTTCCCTTGCAGCTCAAGACAGCTGTCTGGGCTGTTC
TCTACTACTTCTATACGGGCCTGGGAATCACAGCTGGATACCACCGTCTTTGGGCTCACTGCTCCTACT
CTGCCACTCTTCCTCTCCAGATCTTTCTCGCTGCCGCTGGCGGTGGTGCCGTCGAGGGCTCCATCCGCT
GGTGGGCTCGTGGCCACCGTGCTCATCACCGTTACACCGACACCGACAAAGG
