# Supplementary material for: Antibody screening reveals antigenic proteins involved in Talaromyces marneffei and human interaction
Source: Front Cell Infect Microbiol. 2023 Jun 19;13:1118979. doi: 10.3389/fcimb.2023.1118979 (PMC10315666; doi:10.3389/fcimb.2023.1118979)

Epitope prediction

Highlights in yellow depict epitope regions predicted by the Bepipred 2.0 tool. Highlights in red fonts show epitope regions predicted by the ABCpred tool (shown only score ≥ 0.8). Epitope regions predicted by the SVMTriP tool are shown in bold and underline (shown only recommended sequences).

Bepipred 2.0
http://tools.iedb.org/bcell/

ABCpred (score ≥ 0.8)
https://webs.iiitd.edu.in/raghava/abcpred/ABC_submission.html

SVMTriP (Only flag = highest score and recommended)
http://sysbio.unl.edu/SVMTriP/prediction.php

P3 TmHam13

>XP_002149306.1 conserved hypothetical protein [Talaromyces marneffei ATCC 18224]
MGAKKSAVNRPMNAQQRDADISTKLQIYGIYSAFANGKLPSNKQCDVALNSAIKSKWLSSPPKELSEDGR
TLVKDLRDVIDKTKLLFLTKNEGELIQEFIWDAQQITGEEFQRITGPVSKESARGDADQAAEGFKTLGTL
LITNGEFRKLLSDAVTLLRDIAGDTASKAASKLRPDEDALAQIDQPAEENVWHDKPDLNKESLKAQFKEQ
TDRFKPASKQDVQEAANAATTAATGGQQDASVSQIDARAGVNAAKETLQQRAEQNVAPEDRDQVRQVTEQ
AQAVSSEYNRRIKDFLASKMPKERREQIVWRLKKMIVEIQGHSDYQQAIETLLSLAESYAGHGRDISSQG
TTATKGARETTIIHKAETNLRILIERFANSTSTDDFFDSLNTIYRDADQDPRLKEWFRGVDTYIRKCLRE
QGFIMQDEANDQWNKLYDEGRFLLRDRYRSHTDRIADEAKFLATQFDEDPQNRAFRQSLERLFKDLGQDQ
YGKPTFKPHLIKDITNVIVPEIFENASYIP**IPRIEVSDPAVDMVIENLII**ESDNLMPNVLEFGTDNYWRW
GRKKISSFDDHKVMISASGIQADLRDVSYYFKKKQGFPSLTDIGVMDILLGGSGFGFKIAASKAQKNDHN
AVFKLDSVKVNVKNLTIKLKKSKHKMLFSIFRPMLLNVVRPVLEKVLEAQIREAFQKADAFAYQVQTEAQ
RAQETMREDPENAKNIFARYADATRHVITEKKKQAEAIAERGTKVHMAMTHQDAMFKDIKLPGGVTNKAT
EFKELSAKGDRWQSPVFNWGGASPTSNFPKASAVSRKPHTAAESHLQEKSTDGVNGVGASAVNGLHGVST
TGATARGSSGKAMLPTNGVTNGQSATNGTFQKEVERAFDANGTTLPTLGGV

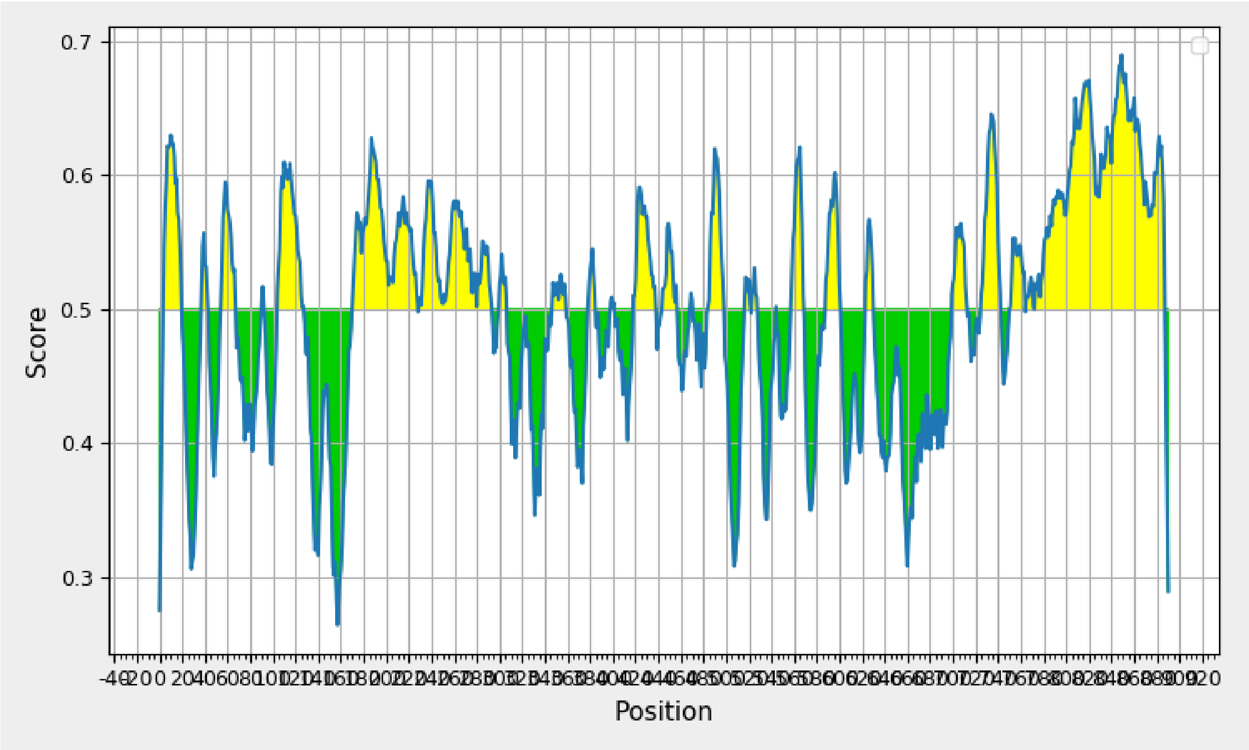

P6 TmMon1

>XP_002144612.1 conserved hypothetical protein [Talaromyces marneffei ATCC 18224]
METANDAPSTTLEGQHEADAQNSRTQQPQQSPDSQEDDAEEVRPPLPPRPETIDLLNEGIAFRTSTARPN
LQSHATTALSLTDITGQTNADGRDGFVAGFGRTLLGRGLRAKASLSQLNSARGSEAGDTASVLSFAPNSE
EGQDESLFGEFANETNAQDISGNIEVLGYDEYPQDGNEYEFVEEFEPIGELDEDGQNEESLLQKWKEKRK
HYLILSAAGKPIYTRHGDSGLVSGYIGIIQTIISFYQDADDTLRSFSAGDTKIVILSKTPLYLVAISRLL
E**SESHLRLQLDALYMQILSTL**TLPALNHLFSIRPSTDLKRPLQGTETLLSSLADSFTKGSPTTLLSALEC
LKLRKAHRQVI**NNILLRNRAEKLLYGLVAAG**GRLVSVVRPKKHSLHPGDLQLLFNMIFEADGVKAGGGES
WIPVCLPGFNSSGYLYMYVSFIDLNDESGGVIMNDDTPKDESVAIVLISADKESFFQLQEMRNKLVGQMR
KSGSLNIMKESIKKGRPSPTDIVPDTVLRHFLYKSKAHVQFVMSAYAPDFTSLTRHRRLISTYNSLHESV
HARNTHVKIHYGTSKSASVFAWVTPIFELYCVAGPDATRTPISQGANKIRQWVQKEEERLFIIGGAVSSI
FHDLKDKRCSNML

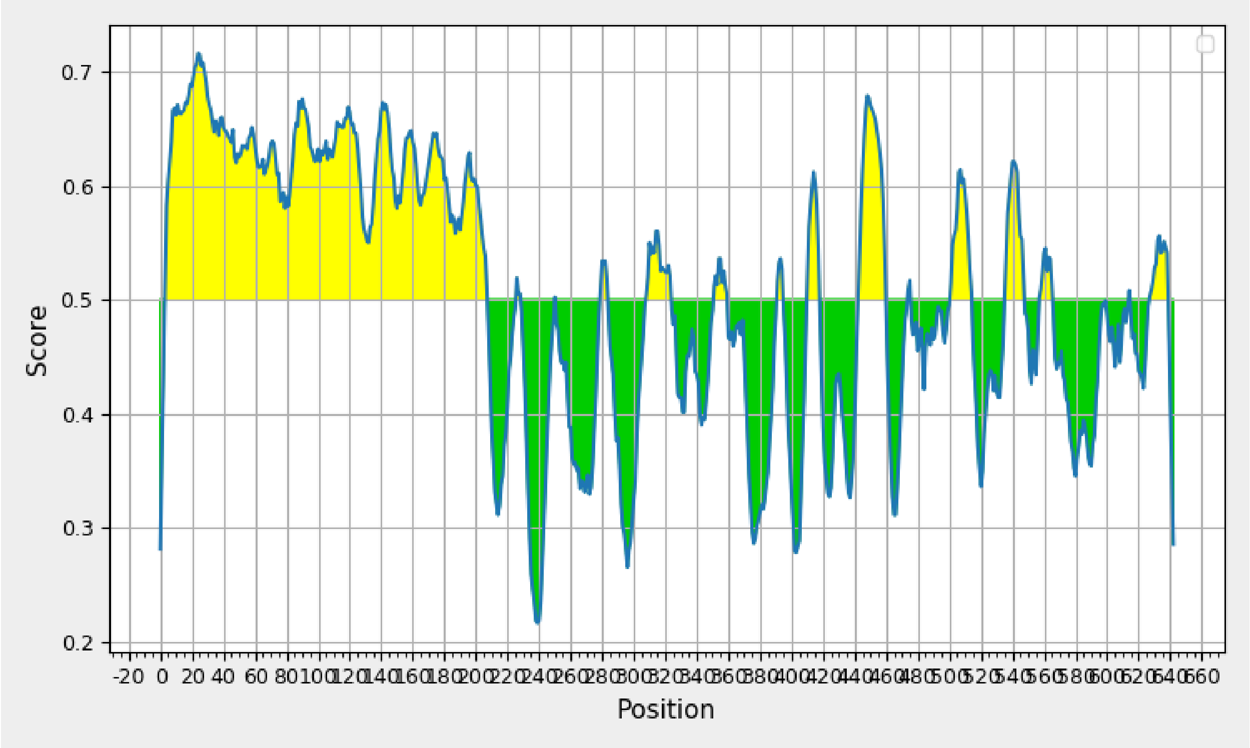

P7 TmFus1

>XP_002146157.1 SH3 domain protein [Talaromyces marneffei ATCC 18224]
MSHAHFHRHERRNFISDVEDFFGVNYAKGPQSTVTETASIVYVTASPTFTGPIGGYITGTDTASPADPTT
TAGKGAPVAQSSTKTTAASSKSDTSTTTKAPAPTTHTTSTLSTTSTSLSDLLTTLSDPPATTFLTSSSTQ
SAS**STSTDTALDQLSSSPTASPT**TSAAASTSNGLTGGAKAGIAIGVLFGVGLIAGLILFWLHKQKKNREE
AAAAAAAAENEKFTPSQPPPMTSAQSPQSMAAYSSAPSTPATAPQVSLRPITQFNPLLSQPGGANPYAAG
AVGAAVGGAAAAAGGLQVNRSAERPYSGSAHVPPQSPRQDPFTDPVNPFDNGAQTASPPMPPAKDASSPV
RDLTPSPTGSAHNLASPIAEEPSAGSVEAAAAGAVAGAAVGAAAVAAAAAKSDDKPAESRTPSPEYVENA
GSRPQSPVVGGAGAPVSN**VHRVQMDFTPSLADEMELRA**GQLVRLLKSYDDGWTQCSSMDGSVQGIAPRTC
LSARPLQPRRSPGPNGPGGPRGPPIMAPNGRPMSPAGGRMGPPPQMKGAPPRFYNDGRPMTPTGSGMPQF
PPPPGTPRAMSPGPGRGVDVPRPLTPGGSRPASPAGGRSRSNSASVAQMGGRPAMHRGSQSPLAPPTGPL
PGPPMQPVQSPTAAPAAPAPQVQNDARPEPAIERKPVPGQQ

>>Cannot be identified by Bepipred 2.0
>> Below threshold by Beipred 3.0

P9 Fbp

>XP_002151273.1 fructose-1,6-bisphosphatase Fbp1, putative [Talaromyces marneffei ATCC 18224]
MAANGNGGSGVGQENINTDIITLTRFLTEEQLRVPEATGDFTLLCHALQFA**FKSIAYYIRRASLINLTGL**
AGSSNITGDDQKKLDVIGNDVFIAAMRGSGKVRLLVSEEEEEAIIFDEHPNARYAVVCDPIDGSSNLDAG
VSVGTIFGIFRLPDEILGPNKTVSAKDILRDGTELVASGFTMYGASAQLVITMRGGSVNGFTMENSLGEF
ILTHPKMQLPTKRAIYSVNEGNSMYWDDWVKDYFHSLKYPAEGDKPYSARYIGSMVADAYRTLLYGGIFA
YPADKKSPKGKLRILYECAPMAMVFENAGGLAINSHGERLLT**VLPEHIHDRSGVFLGSKDEV**QKAIDVYN
KHHKK

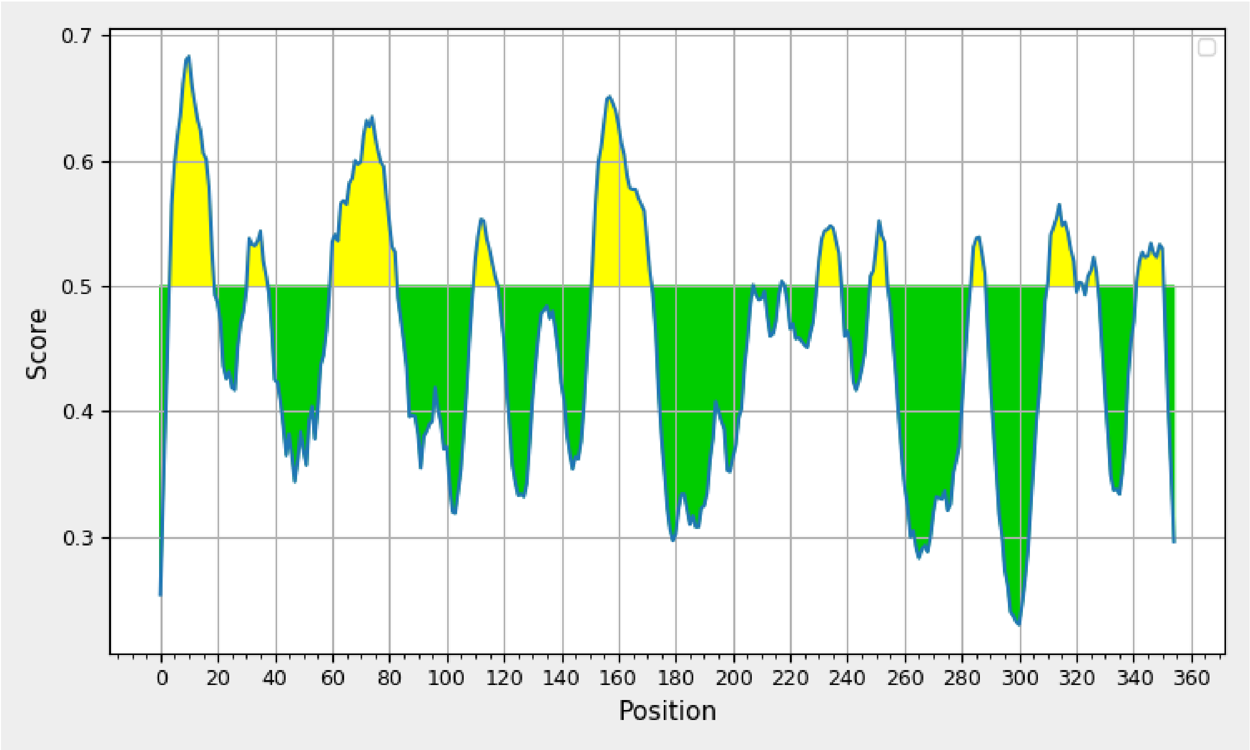

P10 RPL20A

>XP_002150233.1 60S ribosomal protein L20 [Talaromyces marneffei ATCC 18224]
MGRLNEYQVIGRHLPTEANPTPKLYRMRIFAPNTVVAKSRFWYFLTKLKKVKKANGEIVSLNVISEKRPT
KVKNFGIWLRYDSRSGTHNMYKEFRELSRTDAVDSLYQDMAARHRARFGSIHILRVIEIEDNESIRRPYI
KQLLTKGLKFPLPHRVPAQSSKKVFAYKRPSTFA

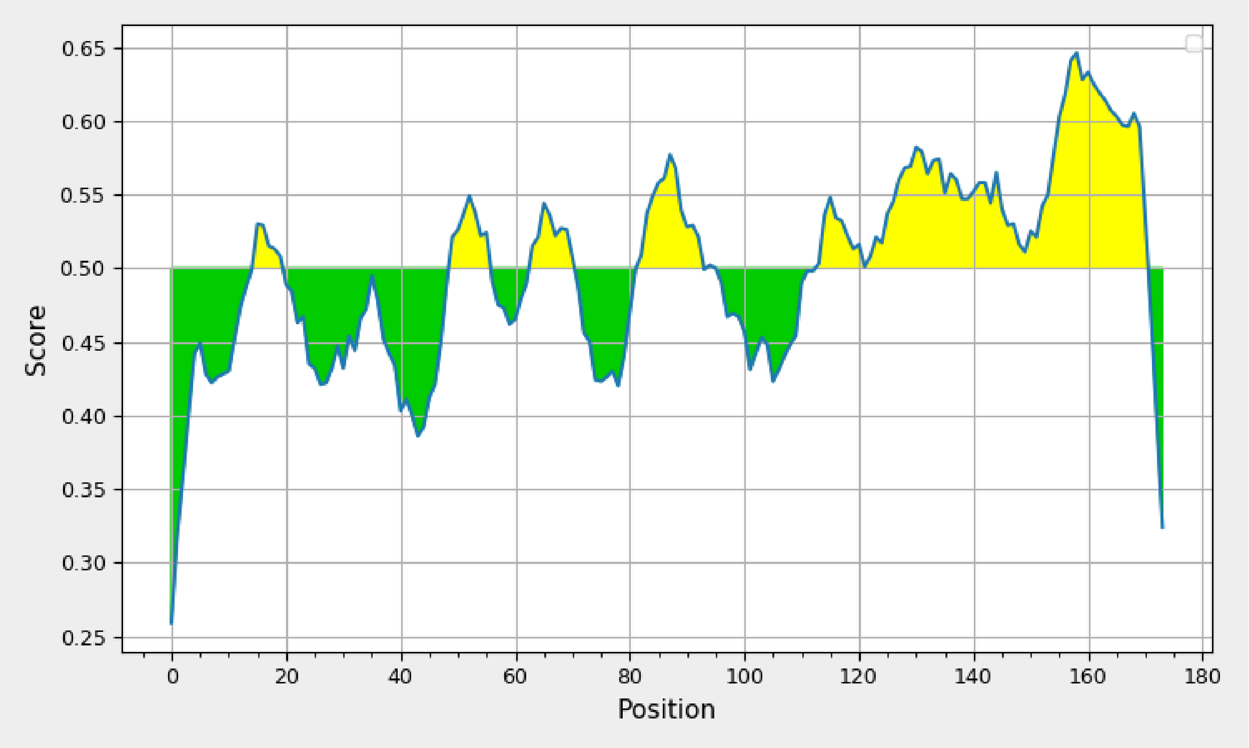

P11 Pho88

>XP_002151103.1 phosphate transporter (Pho88), putative [Talaromyces marneffei ATCC 18224]
MAAQVTNLVIILGMMQVSKRIPFENPDVLLGVRALYIVSNVLILGIYLYVQSKISKKKDLTTLKYVEPAP
MGSGEEPRPVTTTVMEYDKQQLRQLMRSQLMGVGMMAVMHLYFKYTNPLLIQSILPVKSALESNLVKIHV
LGKPATGDLQRPFKAAAGFMGMGQGEI**KSDKASIENAEKNWRGGVKE**E

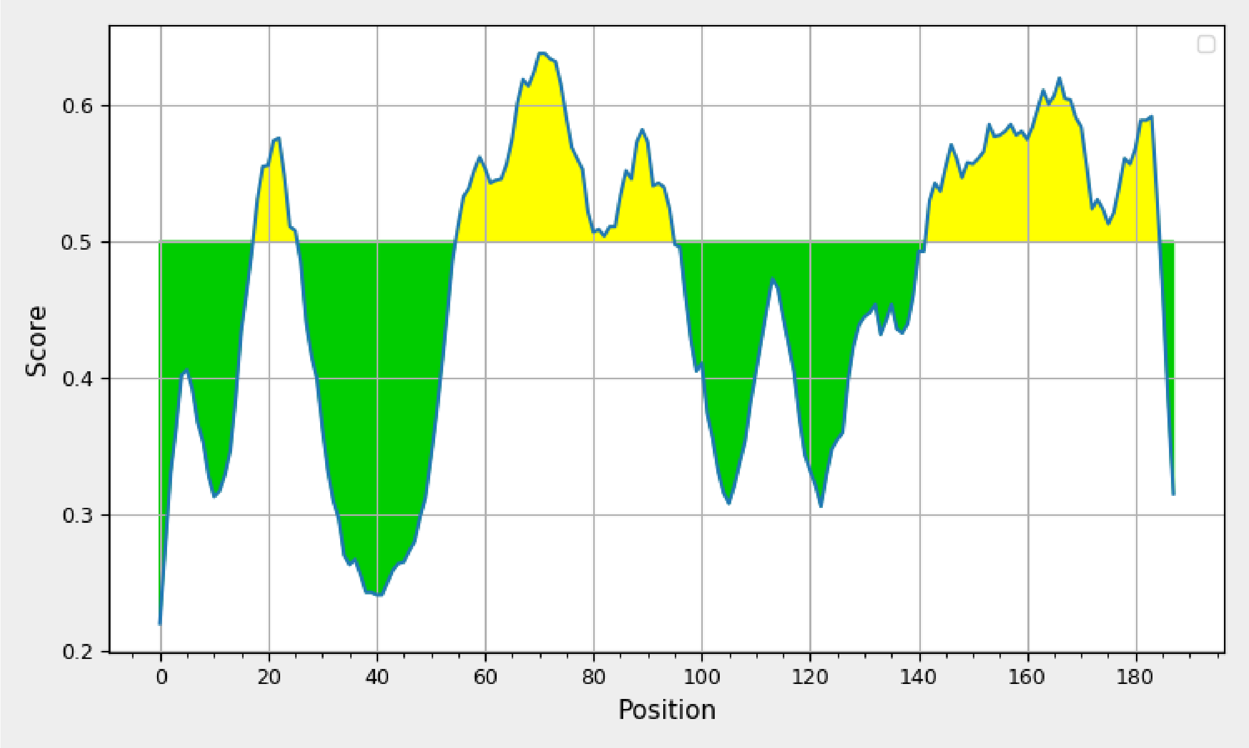

P12 Marvel

>XP_002152818.1 conserved hypothetical protein [Talaromyces marneffei ATCC 18224]
MAMLSSSLSRPILLGIRT**MQWASSVIALGIYAYFVHHQ**RSGTNPIFNLVISVLSVVFFIPAFVSPFMTVL
SKWVALIDMVFSYLWLTAFVLAAQSYNYGDVYLKAPSGVRVSIKHAAESFTFLAFIFT**FFGLLTEVATRW**
TDADDTPVTREKHNGDTRAPLDAPANPTGPAAV

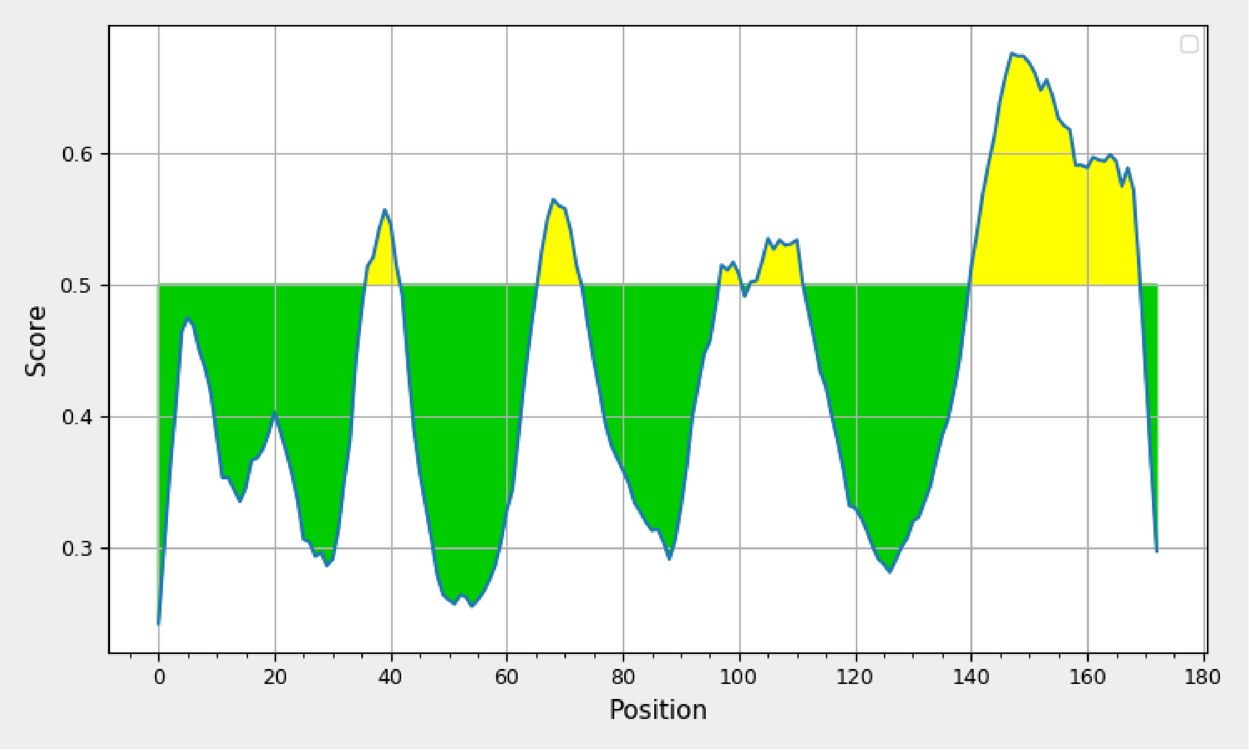

P13 Nuo21.3

>XP_002144515.1 NADH-ubiquinone oxidoreductase 21 kDa subunit, putative
[Talaromyces marneffei ATCC 18224]
MSREVAKAAKSASNAIAVSKKYTVQSTGIWEVIRRTLAVDPTRSTGVPLNSQFRNPAPGALPPQSYDEPV
TLPAADLADNPYWKRDVRRNYPQLSVFSQGDVAGLLTFGNKQAPKED**ALQLGEAGEKQLIAAKQEGD**EKG
L**AAQFEKDKNSVKSVLGPDGL**PPLPVRLNNLAKYELGSGQGYPEKYPCRTFV

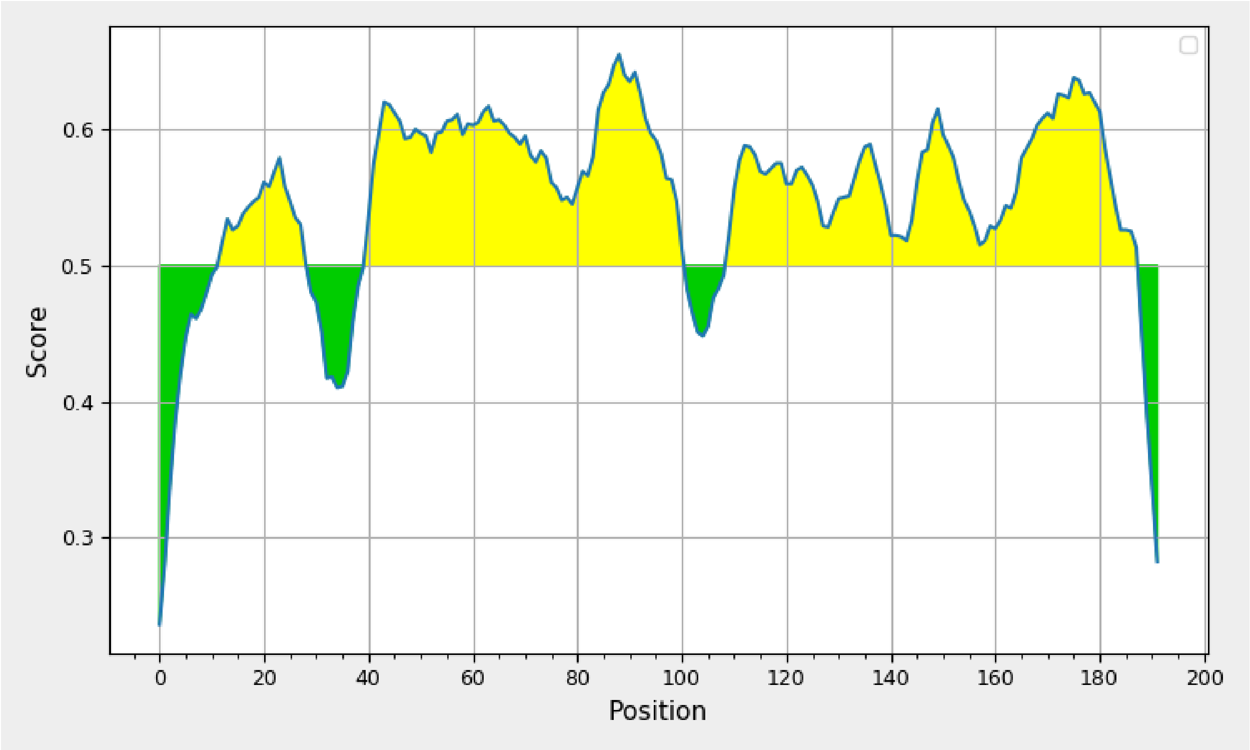

P14 C2H2 zinc finger protein

>XP_002150081.1 C2H2 finger domain protein, putative [Talaromyces marneffei ATCC 18224]
MSSYPKYNSYRFPNSQNEENSRRNHHPAQQQQQQQPVQPPRGYMEANYSWNNENTNRSYLNTNNAYSRAQ
DSLSSFPHFPVELSRYSQHGTRTDENTQRSAGLYRYPSQSNSAVNAQATTTYNQQPQHVSNAVSLTSLAS
YTYRNTSVNNAAQPKWRPSPKANQRGQRNTENDKSPLVTQPSTNTSLNARSYSQMSHGYSLYTTNTSSVA
NPSHTVISIETLPTVTQDYPQVSETRTTDQISSSNVQYINPTDLYIQQYFLAQERARATEPEQMAGEEAR
KKEVEAQTQVQAEAVARAAEEASSTNAQATPTSTAKGTVKPKPAKNTSKKRSSAKKDAAPADPLDPEGDH
DMALEMRMMLEKLHSMRSKDPSLFAKLWDDFKNPAASTQTTATSEQAAAGPSQSTTNPPPSTRTLSKTPK
GQAQAQDGFPDLGKFPAQRRKREKQSDVAEVGAANSQLDASAPVSQVNVNSMRNQQQHQSPAPVPASVAK
KTAAKSLQKASTAASSASKPKATPAQEATWPVATQQKLAKAVSGYLSKGAANNGKECSPESLMLLLRNNP
TYPDLCAQLESRGFVLDRQAMARFLLVAVPALLSGKNGQEKKAEAIKNTPSAQASRSLPPTMTQPPPHPP
SLPPQPVQPTQASQLVPPAQSTIPPRPALPQGVPTSDLDVVYYQPGAPAHKPHAPKNVDPTISTARPQSN
QKGKTSWQRPRRSDGAWISNHPVGPKAAMAR**KRFFSEIVDMSQASSDEEGI**PSDDDDDDEDGFSGRPNAS
MNMSISQPPNFPDSGPDPMDIDTDSPYPIEQDLSDSHAAIPEPDIEFNPDDESVLCEIKRLPNITKPLNP
SDALKRVYYNPKSIARDIMLATGRHPSERPLNFHLMHFTQTFSGVTVRSDLETFKWNLVDPGGPSMPVVE
LEDILVEPPEFTRKKRRRRDVGSRDDADVKGPTSESQPQPSGTVATPGQPLSTSVSTPHGVRDSMVGTPT
TGQRTGRRGRPPGAKNKNPTKATLKALVKTTAGSSTRDAQPAVPAPAPATSVPELSYPMFTCEWASCPAQ
HDVHTLERHVVKNHISGQTTCLWQNCP**NLATEYSGEGLKEHLAQAH**IQPLAWKYGDGASVNGNVQLDLDR
YLNANGLIVTPDAITAGENDALIFPVEPIPIRAFNKLYGDQKATDRARQVLRAVQKRRRRVGIGLEQEGC
EFSTPVRNKLFVNDEEFYEVVTDGEQGTDDWFSQGETY

>> cannot be identified by Bepipred 2.0

P17 Gpx1

>XP_002152894.1 glutathione peroxidase Hyr1, putative [Talaromyces marneffei ATCC 18224]
MASATTFYDFSPPDKKGNPYPLTDYKGKVVLVVNTASKCGFTPQFAGLEKLYKSIEAKHPGAFTILGFPC
NQFGNQDPGSNDEIQSFCQVNYGVTFPVLGKIDVNGSKAEPLFEWIKSEKPGLLGVKRVLWNFEKALING
KGEVVGRWRSITKPES**LEATILKEIDIASKDVKGVE**VVPTATETAASAAPAEEAKEA

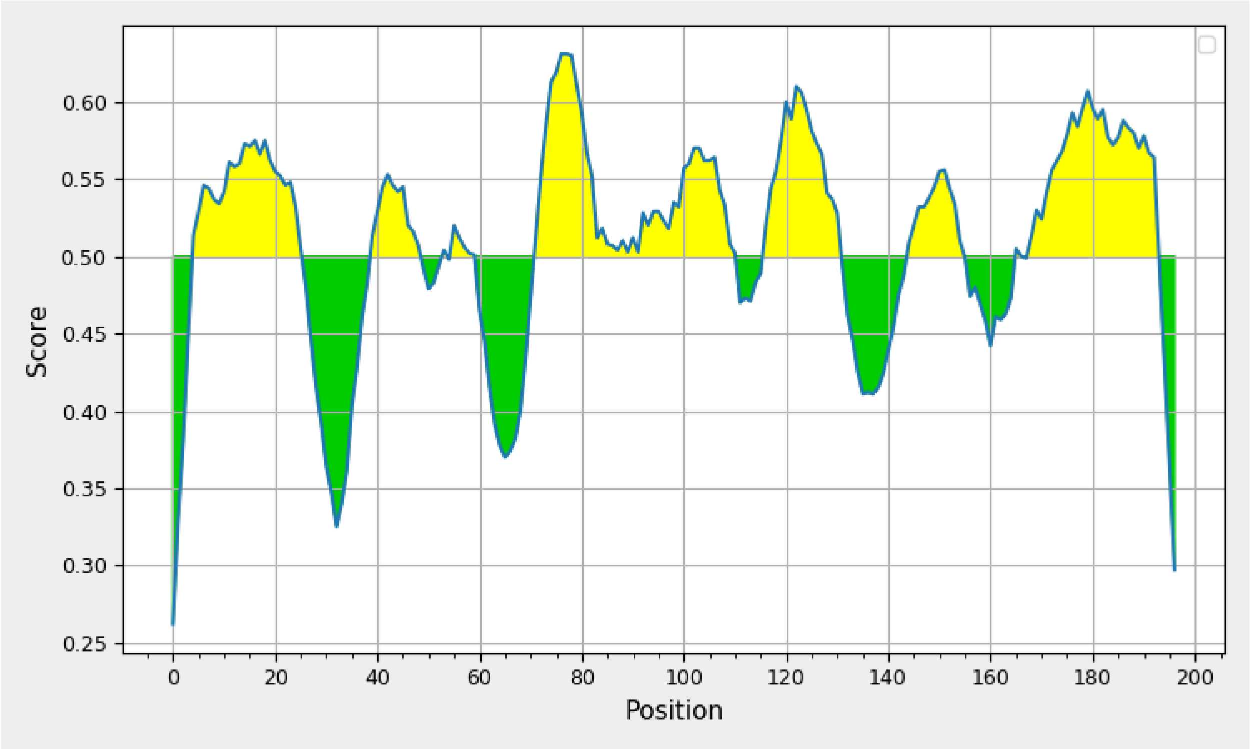

P21 TmNbr1

>XP_002152652.1 ZZ type zinc finger domain protein [Talaromyces marneffei ATCC 18224]

MSDLVPLSKSSAILSTSMATPSPSAPAVGPDTLITIKIL**HNDSVNRRFKIPLRDLGARV**FPQKVRYLLAV
APTDSLVLERYSDSLANYIVLDSENPAVYKQLYRAAKAKLKLRIKATTKPQESTDSPVVTAPIEEPRIQE
QSHQRFRYLETVLS**PSTTASAQTKNMTSPLTSRA**RAFSPVSSVHEKQQAFPLRFSDNNYLGNAFCIDCNN
CGGSIPSEHYHCGICDDGDYDLCLNCVDAGVSCPGDDHWLL**KRVVQNGVIINSVTEIAPKR**LGSLEPVAT
EKPAPVKEEPEEEPKVEEKEAIVPEPEVKEEPKEVADERTCNACFREFSESSMVHCDNCEDYDLCIGCLL
KNSHGHNPAHAFSIIQENQLGLKNLVLSRCRPGRHYHHAAICDGCEKRIVGVRHKCLSCPDWDYCWSCAK
TADQSHPQHRFVPIYGPISEYSFSQDVHYGIYCDGPLCRGKPSTSYITGVRYKCAVCHDTDFCAACEALP
TNTHNQTHPMIKFRTAVRNVTVNTLGDDGFGGQTMVMGDRTPPAVRSSANVTPTSPVEAPSVVEKVEPEA
VKEETAEEEPAPETPETPATPRRSSVDFTDDYSAYFMKDTVSDGTAMAPSQVFQQTWTLYNPGPTTWPVG
TSVRYVGGDAMFNINTEHPSSVVALAVAMSSNELVHPVAPSESADFSVTLKTPQRIGSSISYWRMKLPNG
TPFGHKLWCDVKVVEQPIAATEPVVPVVEEEAESVVSEYESATVVDAATETESEAEMAGSNMVFPKLEKE
SPVSSTHTLAQNPAPSYTAPSVDDQALPD**DMESLTLEDSDDDGFFTDEE**YDILDASDQESINGKH

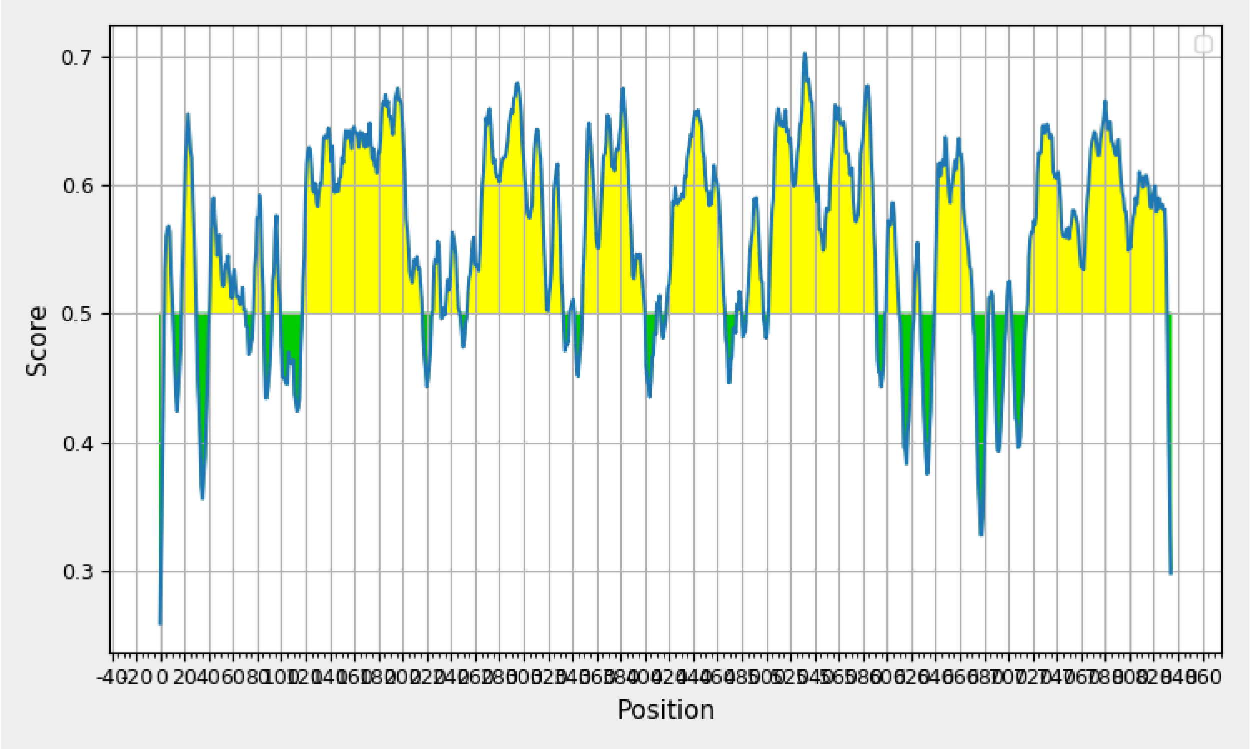

P24 Nmt1

>XP_002145167.1 thiamine biosynthesis protein (Nmt1), putative [Talaromyces marneffei ATCC 18224]
MGFKAMIHTLAAKARNFPVTSFGSLLDEPFTGVVYLKDSGITTDFKTLKGKRIGYVGEFGKIQIDELTKY
YGMTPDDYTAVRCGMNVTKAIIEGNIDAGIGLENVQMVELEEWLAAQGRPRDDVQMLRIDQLAELGCCCF
CSILYIAND**SFLAANGDKVMKFLRAIKRA**TDFVIAEPKKAYEE**YVDFKPIMGTEVNRKIFERS**YAYFSRD
LKNVPRDWQKVTNYGKRLGILDASFQPNYTNELLTWDLDADSTDPIGDQQRMAELQKKVATEGGFQRLEI
AA

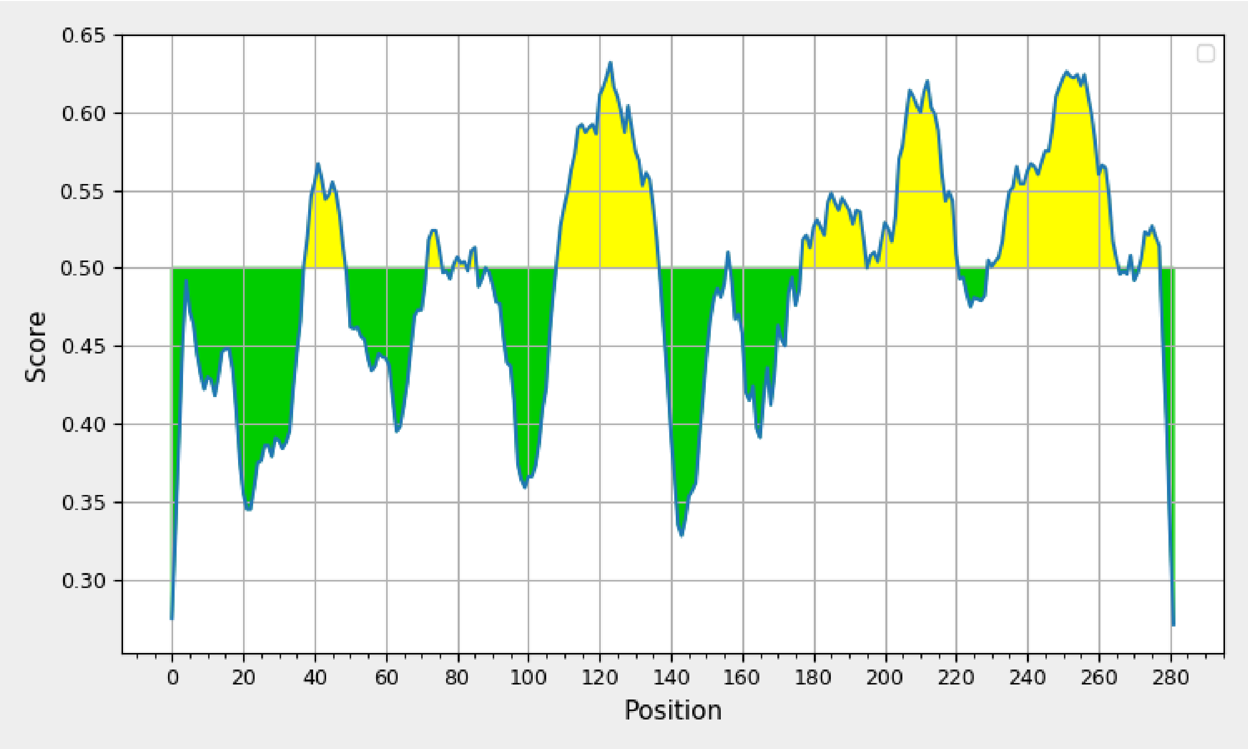

P26

>XP_002153087.1 conserved hypothetical protein [Talaromyces marneffei ATCC 18224]
MRSILISAALIGSALAYPGGYWAERRHYGTGIGTGTQPHPPPQTSTLTTTSMPPASTTTTTTSVSEWSTV
TETETNTKTVFVPCSTS**VGTRGSSTVYSTWLTTTTSV**WTTTHTTTVPVYGTTTTPVGGVGPVGSETGLAC
PLPVTTTHTETKTTTATVTVTVAVTAPGAPETTTTKPAVAIPPPPPPPPVESTSTSTLTHTSTSTATATA
TTTATGTETSVEPTSTHTTTTTKPASTTTTITTHLGTGTITGPVGTGTGSPSWVHKRNEMKKPIIHRVL

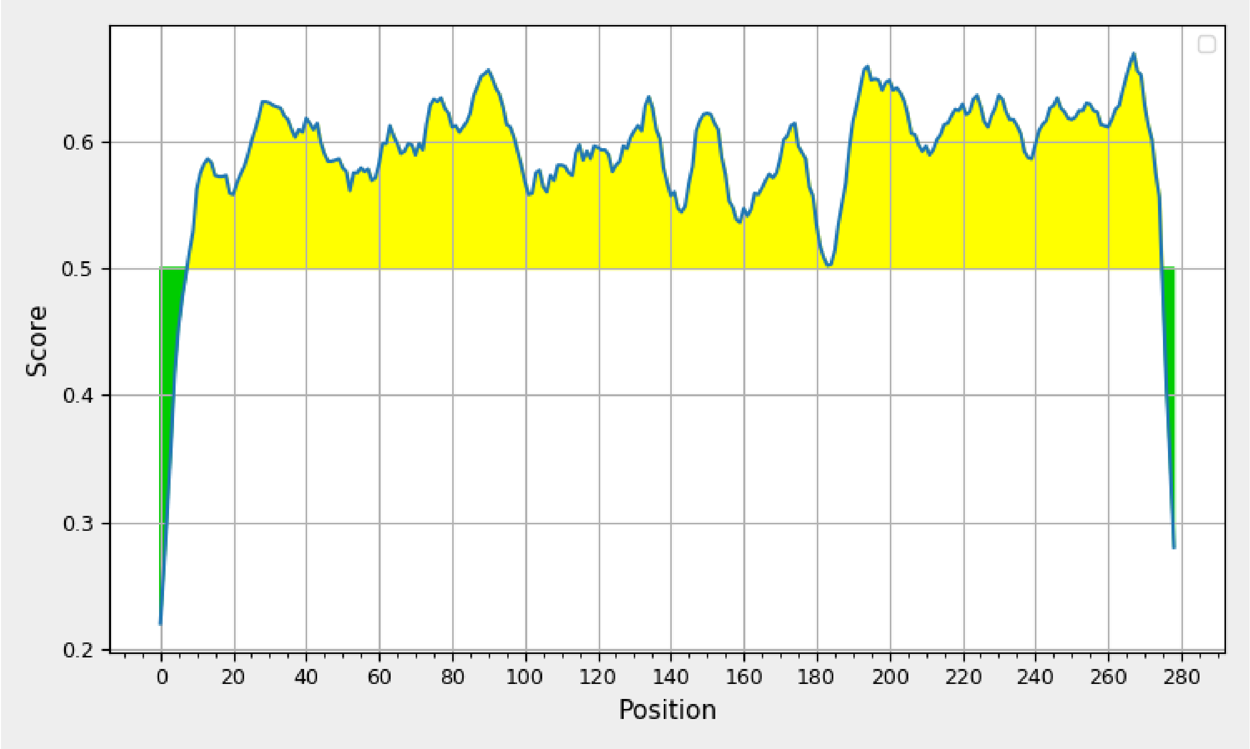

P28 SdeA

>XP_002152122.1 stearic acid desaturase (SdeA), putative [Talaromyces marneffei ATCC 18224]
MSATTADARPRPAADTKKVHIADTKMTLKNWYKHVDWLNVYFIIGIPLYGCIQSLWVPLQLKTAVWAVLY
YFYTGLGITAGYHRLWAHCSYSATLPLQIFLAAAGGGAVEGSIRWWARGHRAHHRYTDTDKDPYSVRKGL
LYSHLGWMVMKQNPKR**IGRTDITDLNEDPIVKFQHR**HYIKIVAFMGLVFPMMVAGLGWGDWWGGFVYAGI
LRIFFVQQATFCVNSLAHWLGDQPFDDRNSPRDHVITALITLGEGYHNFHHEFPSDYRNAIEWHQYDPTK
WFIWTCKQLGLAYDLKQFRANEIEKGRLQQLQKKLDKRRTQLDWGIPLDQLPVMEWDDYVEQAKNGRGLI
AIAGVIHDVTDFIKDHPGGRAMINSGIGKDATAMFNGGVYFHSNAAHNLLSTMRVGVIRGGCEVEIWKRA
QKENTNVDYVRDTNGQRVVRAGEQVTKIPQPVATADAA

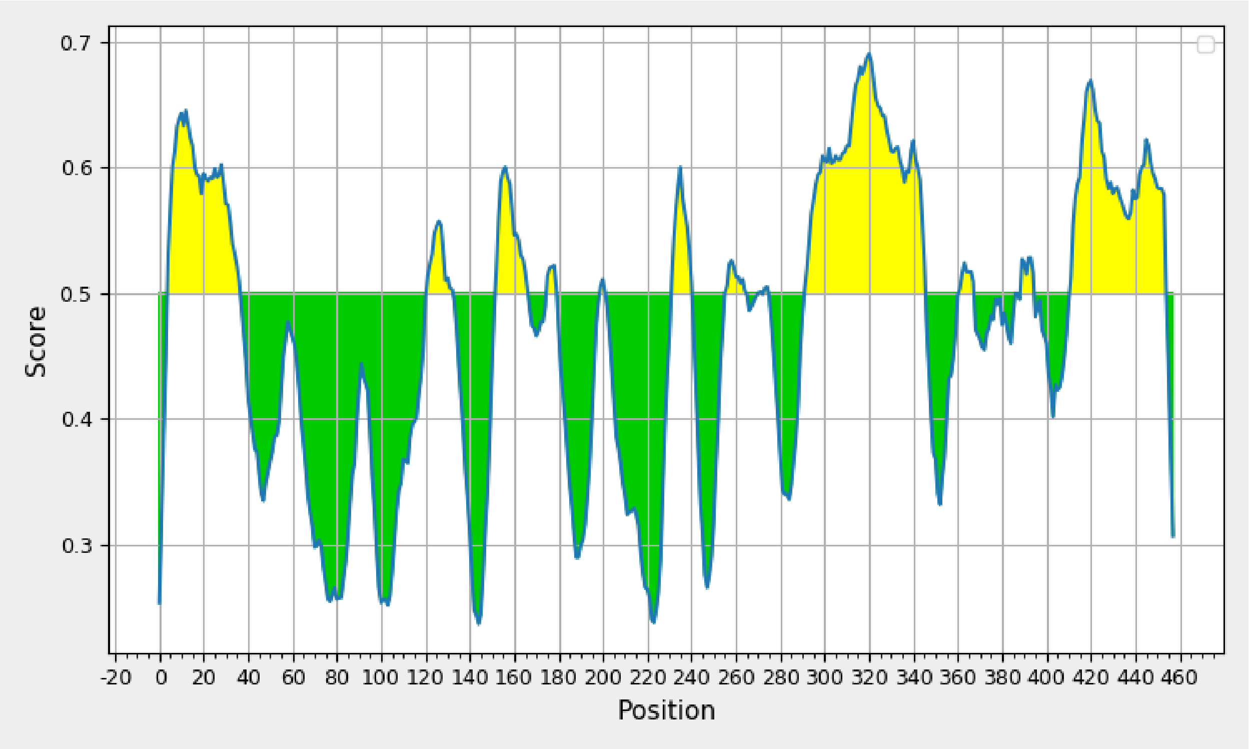

Supplement: Supplementary file 5 [file DataSheet_2.pdf]
